# Novel Feature Selection and Voting Classifier Algorithms for COVID-19 Classification in CT Images

**DOI:** 10.1109/ACCESS.2020.3028012

**Published:** 2020-09-30

**Authors:** El-Sayed M. El-Kenawy, Abdelhameed Ibrahim, Seyedali Mirjalili, Marwa Metwally Eid, Sherif E. Hussein

**Affiliations:** 1 Department of Communications and ElectronicsDelta Higher Institute of Engineering and Technology (DHIET) Mansoura 35111 Egypt; 2 Computer Engineering and Control Systems DepartmentFaculty of EngineeringMansoura University 68779 Mansoura 35516 Egypt; 3 Centre for Artificial Intelligence Research and OptimizationTorrens University Australia 386703 Fortitude Valley QLD 4006 Australia; 4 Yonsei Frontier Laboratory (YFL)Yonsei University 26721 Seoul 03722 South Korea

**Keywords:** COVID-19, CT scans, convolutional neural network, guided whale optimization algorithm, features selection, voting ensemble

## Abstract

Diagnosis is a critical preventive step in Coronavirus research which has similar manifestations with other types of pneumonia. CT scans and X-rays play an important role in that direction. However, processing chest CT images and using them to accurately diagnose COVID-19 is a computationally expensive task. Machine Learning techniques have the potential to overcome this challenge. This article proposes two optimization algorithms for feature selection and classification of COVID-19. The proposed framework has three cascaded phases. Firstly, the features are extracted from the CT scans using a Convolutional Neural Network (CNN) named AlexNet. Secondly, a proposed features selection algorithm, Guided Whale Optimization Algorithm (Guided WOA) based on Stochastic Fractal Search (SFS), is then applied followed by balancing the selected features. Finally, a proposed voting classifier, Guided WOA based on Particle Swarm Optimization (PSO), aggregates different classifiers’ predictions to choose the most voted class. This increases the chance that individual classifiers, e.g. Support Vector Machine (SVM), Neural Networks (NN), k-Nearest Neighbor (KNN), and Decision Trees (DT), to show significant discrepancies. Two datasets are used to test the proposed model: CT images containing clinical findings of positive COVID-19 and CT images negative COVID-19. The proposed feature selection algorithm (SFS-Guided WOA) is compared with other optimization algorithms widely used in recent literature to validate its efficiency. The proposed voting classifier (PSO-Guided-WOA) achieved AUC (area under the curve) of 0.995 that is superior to other voting classifiers in terms of performance metrics. Wilcoxon rank-sum, ANOVA, and T-test statistical tests are applied to statistically assess the quality of the proposed algorithms as well.

## I. Introduction

Coronavirus (COVID-19) is a virus infection, named Severe Acute Respiratory Syndrome-Corona Virus-2 (SARS-CoV-2), which appeared in Wuhan toward the end of 2019 [1], [2]. Due to the outbreak, COVID-19 has emerged as a pandemic that threatened human lives and caused devastating economic consequences that arose since that time. Therefore, a significant number of researches were instantiated to discover a solution to control the spread and mortality. Due to COVID-19 implication, many research proposals were conducted to assess the presence and severity of pneumonia caused by COVID-19. Such studies are centered around the screening process to discover early-stage patients, the proposed treatment protocol, and the assessment for various stages and recovery of treated patients. The image modalities including Chest X-ray and Computed Tomography (CT) are non-invasive and are widely used in hospitals to detect both the presence and severity of COVID-19 pneumonia [3], [4]. Compared to CT, even though X-ray is more accessible in hospitals around the world, X-ray images can be considered less sensitive than CT scans for the investigation of COVID-19 patients. [3] reported that X-ray was diagnosed to be normal in both early and mild stages. On the other hand, CT images enable the non-destructive 3D visualization of internal structures and are considered as a powerful analysis tool [5], [6] that has been applied widely to clinical diagnosis [7] and biomedical imaging [8]. In addition, CT has always aimed to achieve improved scanning efficiency in both time and radiation dose [9]. The development of Multi-slice CT (MSCT) has been successful to improve the efficiency of scanning by simultaneously increasing the number of scanned slices [10]. Moreover, dual-source CT managed to achieve a larger temporal resolution improvement, [11].

Machine learning algorithms have been gaining momentum over the last decades for medical applications such as computer-aided diagnosis to help physicians for an early diagnosis, which can lead to better-personalized therapies and enhancement of the medical care offered to patients [12], [13]. Convolutional neural networks (CNN), as a subset of machine learning algorithms, is a unique structure of synthetic neural networks used for image classification. There are several CNN models including AlexNet [14], VGG-Net [15], GoogLeNet [16], and ResNet [17]. In the CNN models, classification accuracy correlates with the extended number of convolution layers [18].

Optimization is the process by which the best possible solution is found for a particular problem from all the available solutions [19]. One of the most powerful methods to solve applications in radiology problems are Meta-heuristic algorithms. The inspiration of most of these algorithms is from physical algorithms’ logical behavior found in nature. The acceptable solutions found these optimization techniques are typically obtained with less computational effort in a reasonable time, [20]. The early diagnosis of coronavirus can significantly limit its wide-spreading and therefore increases the patients’ recovery rates. So, several artificial intelligence (AI) techniques have been proposed for the early detection of COVID-19 in the literature.

In this article, a framework for COVID-19 classification is proposed based on three cascaded phases. The first phase automatically extracts features from the training CT images by a CNN model named AlexNet. Then, a proposed feature selection algorithm, using Stochastic Fractal Search (SFS) and Guided Whale Optimization Algorithm (Guided WOA) techniques, is applied to properly select the valuable features. The LSH-SMOTE (Locality Sensitive Hashing Synthetic Minority Oversampling Technique) is used in the second phase to balance the extracted features. The last phase classifies the selected features by a proposed voting classifier, using Particle Swarm Optimization (PSO) and Guided WOA techniques, by aggregating the Support Vector Machine (SVM) [21], Neural Networks (NN) [22], k-Nearest Neighbor (KNN) [23], and Decision Trees (DT) [24] classifiers to improve the ensemble’s accuracy.

Two kinds of CT datasets are used in the experiments to test the proposed framework. The first dataset has COVID-19 CT images, while the second dataset has extra CT images with clinical cases that have no COVID-19. For feature selection, the proposed (SFS-Guided WOA) algorithm is compared in experiments with binary versions of the original WOA [25], Grey Wolf Optimizer (GWO) [26], Genetic Algorithm (GA) [27], PSO [28], hybrid of PSO and GWO (GWO-PSO) [29], hybrid of GA and GWO (GWO-GA), Bat Algorithm (BA) [30], Biogeography-Based Optimizer (BBO) [31], Multiverse Optimization (MVO) [32], Bowerbird Optimizer (SBO) [33], and Firefly Algorithm (FA) [34] in terms of average error, average select size, average (mean) fitness, best fitness, worst fitness, and standard deviation fitness. Lastly, the proposed voting classifier (PSO-Guided WOA) result of 0.995 is compared with voting WOA, voting GWO, voting GA, and Voting PSO in terms of Area Under The Curve (AUC) and the Mean Square Error (MSE). The main contributions of this article are as follow:

• A COVID-19 classification framework based on proposed algorithms for feature selection and classification is developed.
• A novel feature selection algorithm based on SFS and Guided WOA techniques is proposed.
• A novel voting classifier based on PSO and Guided WOA techniques is proposed.
• The proposed framework can classify the input CT images to COVID-19 or non-COVID-19 effectively.
• The proposed framework is evaluated using two datasets of COVID-19 CT images and non-COVID-19 CT images.
• Statistical tests of Wilcoxon rank-sum, ANOVA, and T-test are carried out to ensure the quality of the proposed algorithms.
• This framework can be generalized to the applications of biomedical imaging diagnoses.

This article contains the following sections. Related work and the problem definition are discussed in Section II. Section III introduces the materials and methods employed in this research. Section IV presents the model and the proposed algorithms in detail. Section V shows the designed scenarios and results. Section VI discusses the experimental results. The conclusions and future work are shown in Section VII. See Table 1 for a list of abbreviations.

**TABLE 1:** List of Abbreviations

Abbreviation	Explanation
AI	Artificial Intelligence
ANN	artificial neural network
AUC	Area Under the Curve
BA	Bat Algorithm
BBO	Biogeography-Based Optimizer
CNN	Convolution Neural Network
CT	Computed Tomography
DLA	Diffusion Limited Aggregation
DT	Decision Trees
FA	Firefly Algorithm
FS	Fractal Search
GA	Genetic Algorithm
GWO	Grey Wolf Optimizer
KNN	k-Nearest Neighbor
LSH-SMOTE	Locality Sensitive Hashing SMOTE
MLP	Multilayer Perceptron
MSE	Mean Square Error
MVO	Multiverse Optimization
NN	Neural Networks
PCR	Polymerase Chain Reaction
PSO	Particle Swarm Optimization
ROC	Receiver Operating Characteristics
SBO	Bowerbird Optimizer
SFS	Stochastic Fractal Search
SMOTE	Synthetic Minority Over-sampling Technique
SVM	Support Vector Machine
WOA	Whale Optimization Algorithm

## II. Related Work

In this section, the recent literature utilizing the CT scans for diagnosing COVID-19 patients will be summarized. Then, the recent evaluation of Artificial Intelligence (AI) against COVID-19 based on the CT scans will be discussed as well.

### A. COVID-19 and CT Scans

Recent study proposed several COVID-19 detection paradigms. In [35], Li *et al.* proposed a methodology to recognize the infection rate using the coronal and axial view of lung CT scans. The proposed work achieved a specificity of 100%, AUC of 0.918, and sensitivity of 82.6%. Another study by [36] evaluated COVID-19 disease using visual inspection. They claimed that visual inspection can help to correctly identify the infection. In [37], Panwar *et al.* proposed a scheme to evaluate the lung CT scans and implemented visual inspection-based detection. Their scheme could achieve Specificity of 94%., AUC of 0.892, and Sensitivity of 83.3%. In [2], Wang *et al.* investigated 90 patients’ lung CT scans. Their investigation managed to detect the severity based on the time since the patient got infected. In [38], in addition a diagnostic methodology was proposed based on the CT scans image features. They concluded that the combination of both image features evaluation and clinical findings can early detect the presence of COVID-19. In [39], Bai *et al.* investigated the patient’s information and considered the CT scans and RT-PCR for the examination. They achieved a specificity of 100% and a sensitivity of 93%. In a similar study [40], authors clinically evaluated patients with both CT scans and real-time RT-PCR with an early detection accuracy of 90%.

### B. Artificial Intelligence for COVID-19

Recent works show that the CT scans are mainly utilized to offer fast diagnostic methods to prevent and control the spread of COVID-19 and assist physicians and radiologists to correctly manage patients in high workload. Authors in [41] developed a method based on deep learning to accurately assist radiologists to identify COVID-19 patients using CT images. They used deep learning to train a neural network to screen COVID-19 patients based on their CT images. The proposed method achieved a specificity of 61.5%, sensitivity of 81.1%, AUC of 0.819, and accuracy of 76%. In [42], Ardakani *et al.* proposed a method to diagnose COVID-19 using an AI technique based on CT slices and ten convolutional neural network models to correctly diagnose COVID-19 from non-COVID-19 groups. The authors found that both ResNet-101 and Xception have achieved the best performance. Moreover, ResNet-101 managed to detect COVID-19 cases with a specificity of 99.02%, Sensitivity of 100%, AUC of 0.994, and Accuracy of 99.51%. On the other hand, Xception achieved a Specificity of 100%, Sensitivity of 98.04%, AUC of 0.994, and Accuracy of 99.02%. The authors recommended the use of ResNet-101 to characterize and diagnose COVID-19 infections due to its higher sensitivity.

Another study in [43] used a large CT dataset to develop an AI method that can diagnose COVID-19 and differentiate it from normal controls and other types of pneumonia. The authors investigated the significance of identifying important clinical markers using the convolutional neural network ResNet-18 model. Their proposed method achieved a Specificity of 91.13%, Sensitivity of 94.93%, AUC of 0.981, and Accuracy of 92.49% for COVID-19. In [44], the authors proposed a deep learning neural network-based method named nCOVnet for detecting the COVID-19 based on analyzing the patients’ X-ray images. Their nCOVnet method achieved a Specificity of 89.13%, Sensitivity of 97.62%, AUC of 0.881, and Accuracy of 88.10% for COVID-19. Butt *et al.* [45] used a special type of CNN, namely ResNet-18 to classify CT samples with COVID-19, normal subjects, and Influenza viral pneumonia. They achieved an accuracy of 86.7% with 98.2% sensitivity, 92.2% specificity, and AUC value of 0.996.

Chua *et al.* [46] proposed a model based on the CNN architecture model that was trained from scratch. Their model consisted of five convolution layers utilized as a deep feature extractor. K-nearest neighbor, SVM, and decision tree were fed using the extracted deep discriminative features. The superiority of the SVM classifier was demonstrated with an accuracy of 98.97%, a sensitivity of 89.39%, and a specificity of 99.75%. Another study by Wu *et al.* [47] proposed a weakly supervised CNN that could achieve an accuracy of 96.2% with 94.5% sensitivity, 95.3% specificity, and AUC value of 0.970. A ML-method is proposed in [48] to classify the chest x-ray images into COVID-19 or non-COVID-19 patients. A Fractional Multichannel Exponent Moments (FrMEMs) method is used for feature extraction. A modified Manta-Ray Foraging Optimization based on differential evolution is then used to select the most significant features. The authors’ proposed method is evaluated using two COVID-19 x-ray datasets. The recent AI research for COVID-19 is summarized in Table 2.

**TABLE 2:** Recent AI Research for COVID-19

Reference	Methods	# of samples	# of classes	Type of Images	Sensitivity	Specificity	Accuracy	AUC
X. Wu et al. (2020) [41]	Multi-view deep learning model (ResNet50 based)	495	2	CT images	81.1%	61.5%	76%	0.819
A. A. Ardakani et al. (2020) [42]	Deep learning technique (ResNet-101 based)	1020	2	CT images	100%,	99.02%,	99.51%	0.994
	Deep learning technique (Xception based)	1020	2	CT images	98.04%,	100%	99.02%	0.994
K. Zhang et al. (2020) [43]	AI system (ResNet-18 based)	3,777	3	CT images	94.93%	91.13%	92.49%	0.981
H. Panwar et al. (2020) [44]	nCOVnet, transfer learning, deep CNN	337	2	X-ray images	97.62%	89.13%	88.10%	0.881
C. Butt et al. (2020) [45]	Multiple CNN models (ResNet-18 based)	618	3	CT images	98.2%	92.2%	86.7%	0.996
M. Nour et al. (2020) [46]	Training CNN model, deep feature extraction, SVM	2,905	3	X-ray images	89.39%	99.75%	98.97%,	0.994
X. Wang et al. (2020) [47]	Weakly supervised deep learning framework	450	3	CT images	94.5%	95.3%	96.2%	0.970

The importance of the AI techniques in the early evaluation of COVID-19 and the areas where AI can contribute to the battle against COVID-19 are discussed in [50]. The authors concluded that AI is not fully utilized in COVID-19 because of the possible lack of data or excessive data. To overcome these constraints careful balance must be made between public health, data privacy, and the right utilization of the AI techniques. Furthermore, the need for an extensive gathering of diagnostic data will be extremely crucial to train AI, save lives, and limit the associated economic damages.

Most of the above-discussed studies mainly applied statistical analysis and visual inspection techniques to correctly diagnose COVID-19 infection. A lesser number of applied researches used transfer learning and CNN with CT datasets of coronavirus pneumonia patients, non-corona virus pneumonia patients, and healthy subjects. Therefore, more study needs to be conducted that utilizes AI with properly optimized performance metrics. As per the literature review of this work, it is recommended to use the CT images as a fast method to diagnose patients with COVID-19. The proposed paradigms need to be both reproducible and easily validated to can be quickly integrated into the arsenal of battling the COVID-19 pandemic.

## III. Materials and Methods

This section discuss data sets and methodologies of this research. The datasets, dataset balancing, and the optimization methods of WOA, PSO, and SFS are discussed. The CNN models, classification methods, and ensemble learning techniques are also explained.

### A. Datasets

**FIGURE 1.**
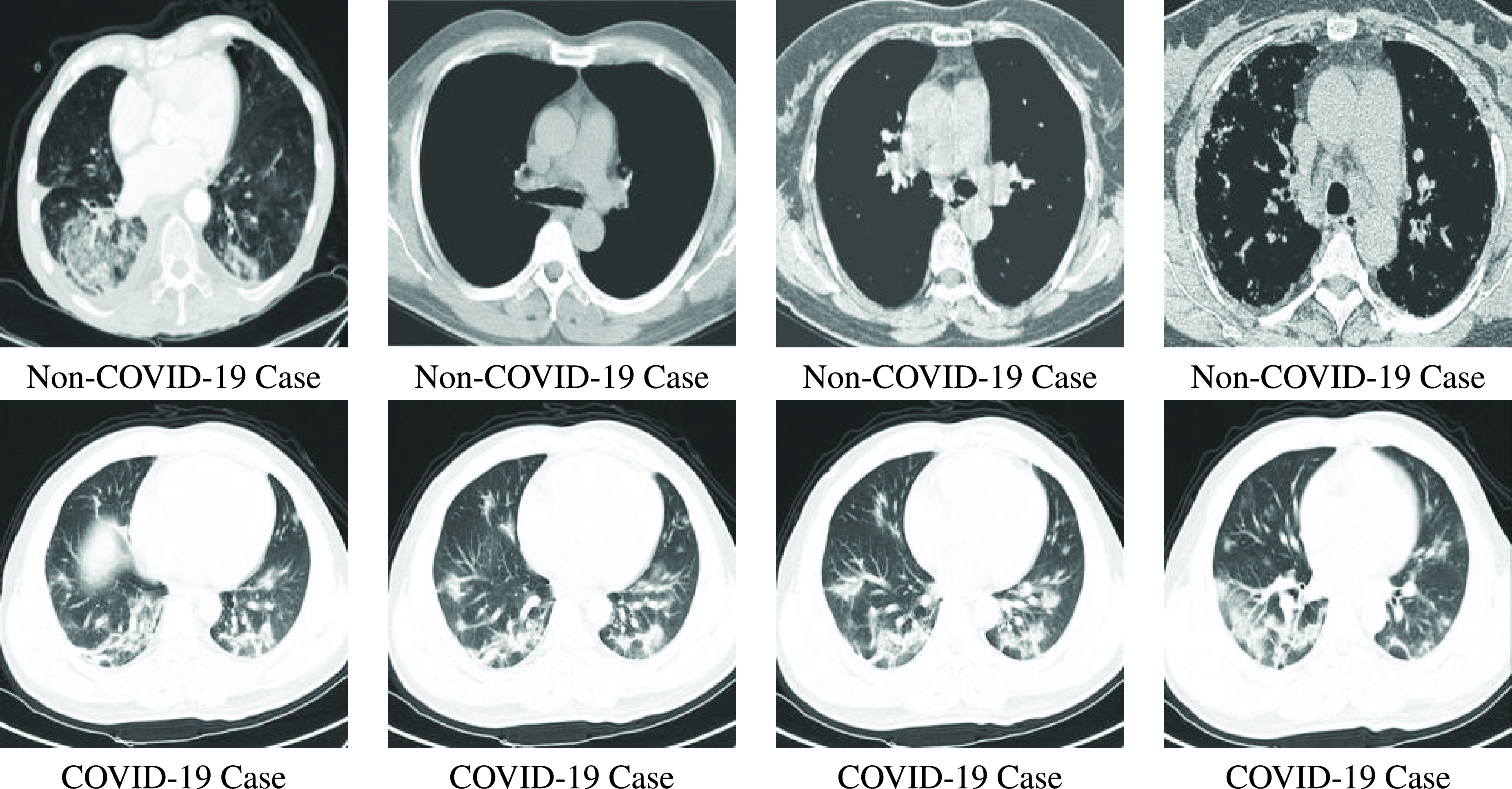
Original images from the dataset for COVID-19 and Non-COVID-19 cases [49].

Data collection is considered as the first and main step in COVID-19 applications. Recently, it has been reported that several data collection works were done on COVID-19. The authors have used two datasets to apply the proposed paradigm. The first is the COVID-19-dataset which has 334 CT images containing clinical findings of COVID-19. While the second is the non-COVID-19-dataset that has extra 794 CT images with clinical cases that have no COVID-19. Figure 1 shows samples of the COVID-19 and the non-COVID-19 cases. The images are collected from COVID19-related articles from medRxiv, bioRxiv, NEJM, JAMA, and Lancet.CTs containing COVID-19 abnormalities were selected by reading through the papers’ figures captions [49]. All patients’ images in the datasets were high-resolution Multi-Detector Computerized Tomography (MDCT) Axial images. The Axial images show bilateral scattered ground-glass opacities with air space consolidation, mainly posterior segments of lower lung lobes with peripheral and subpleural distribution; the picture of atypical pneumonia caused by COVID-19 that is clinically proved by Polymerase Chain Reaction (PCR). PCR is a process that replicates a small segment of DNA, a large number of times, to create enough samples for analysis.

### B. Dataset Balancing

The extracted features from the utilized datasets may suffer from a class imbalance problem. Therefore, several algorithms were investigated to solve that type of problems. Some of the recent algorithms are the SMOTE and the LSH-SMOTE [51], [52]. The SMOTE technique finds its k-nearest minority class neighbors for a selected minority class instance $a$ at random. Then, it randomly chooses another k-nearest neighbor $b$ to be connected with $a$ to form a line segment in the feature space. Euclidean distance is used to sort the instances while selecting the k-nearest neighbors. Finally, a list of k-nearest neighbor’s instances is returned to the main SMOTE class for generating the synthetic instances. LSH-SMOTE was first introduced by [52] to improve the performance of the feature selection SMOTE based optimization techniques. The algorithm starts with hashing and dividing the dataset into buckets by assigning similar items with similar hash codes to the same bucket. That, in turn, can increase the matching probability between similar items leading to a simplified search for the k-nearest neighbors.

### C. Convolutional Neural Network (CNN)

CNN is of the most well-regarded machine learning methods in the literature. One of the reasons of its popularity is due to the automatic hierarchical feature representation in recognizing objects and patters in images [42]. CNNs reduce the parameters of a given problem using spatial relationships between them. This makes them a more practical classifier specially in image processing where we deal with a large number of parameters (pixels), rotation, translation, and scale of images. In fact, CNNs alleviate the drawbacks of Feel Forward Neural networks and Multi-Layer Perceptons by using an alternative to matrix multiplication. We use this powerful method in this study due to the nature of COVID-19 diagnosis from CT images and its high-dimensional nature.

### D. Whale Optimization Algorithm

In the WOA algorithm, the inspiration is from the foraging behaviour of whales, in which bubbles are used to trap the prey by forcing them to the surface in a spiral-shaped [25], [53]. Mathematically, the first mechanism by this optimizer is based on the following equation:

\begin{equation*} \overrightarrow {G}{(t+1)}= \overrightarrow {G}^{*}(t)- \overrightarrow {A}. \overrightarrow {D}, \overrightarrow {D}=|\overrightarrow {C}.\overrightarrow {G}^{*}(t)-{\overrightarrow {G}(t)}|\tag{1}\end{equation*}

where vector $\overrightarrow {G}(t)$ represents a solution at iteration $t$ and vector $\overrightarrow {G}^{*}(t)$ represents the position of the prey. the “.” indicates pairwise multiplication and $\overrightarrow {G}{(t+1)}$ represent the updated position for the solution [54], [55]. The two vectors of $\overrightarrow {A}$ and $\overrightarrow {C}$ are updated in each iteration by $\overrightarrow {A}=2\overrightarrow {a}.\overrightarrow {r_{1}}-\overrightarrow {a}$ and $\overrightarrow {C}=2.\overrightarrow {r_{2}}$ for vector $\overrightarrow {a}$ changes from 2 to 0 linearly and $\overrightarrow {r_{1}}$ and $\overrightarrow {r_{2}}$ are random values in [0, 1].

The second mechanism includes a shrinking encircling, which decreases the values of $\overrightarrow {a}$ and $\overrightarrow {A}$ vectors, and a spiral process for updating the positions as follows

\begin{equation*} \overrightarrow {G}(t+1)= \overrightarrow {D}'.e^{bl}.cos(2\pi l) + \overrightarrow {G}^{*}(t)\tag{2}\end{equation*}

where $\overrightarrow {D}' = |\overrightarrow {G}^{*}(t) - \overrightarrow {G}^{(}t)|$ represents $i$th whales and the best one distance. Parameter $b$ is a constant, represents the spiral’s shape, and $l$ is a random value in [−1, 1]. The WOA mechanism can be simulated by the following equation

\begin{align*} \textstyle \overrightarrow {G}(t+1) = \begin{cases} \overrightarrow {G}^{*}(t)- \overrightarrow {A}. \overrightarrow {D} & if \overrightarrow {r_{3}} < 0.5 \\ \overrightarrow {D}'.e^{bl}.cos(2\pi l) + \overrightarrow {G}^{*}(t) & otherwise \end{cases}\tag{3}\end{align*}

where $\overrightarrow {r_{3}}$ represents a random value in [0, 1].

The last mechanism can be achieved based on the $\overrightarrow {A}$ vector. The position of search agent is updating based on a random whale $\overrightarrow {G}_{rand}$ to allow a global search by the following equation

\begin{equation*} \overrightarrow {G}{(t+1)}= \overrightarrow {G}_{rand}- \overrightarrow {A}. \overrightarrow {D}, \overrightarrow {D}=|\overrightarrow {C}.\overrightarrow {G}_{rand}-{\overrightarrow {G}}|\tag{4}\end{equation*}

Thus, the exploitation and exploration are controlled by $\overrightarrow {A}$, and the spiral or circular movement is controlled by $r_{3}$. The WOA algorithm is shown step by step in Algorithm 1.

Algorithm 1 **Original WOA Pseudo-Code**
1: **Initialize** WOA population $\overrightarrow {G}_{i} (i = 1, 2, \ldots, n)$ with size $n$, maximum iterations $Max_{iter}$, fitness function $F_{n}$.
2: **Initialize** WOA parameters $\overrightarrow {a}$, $\overrightarrow {A}$, $\overrightarrow {C}$, $l$, $\overrightarrow {r_{1}}$, $\overrightarrow {r_{2}}$, $\overrightarrow {r_{3}}$
3: **Initialize** t as the iteration counter
4: **Calculate** fitness function $F_{n}$ for each $\overrightarrow {G}_{i}$
5: **Find** best individual $\overrightarrow {G^{*}}$
6: **while** $t \leq Max_{iter}$ **do**
7: **for** ($i = 1: i < n + 1$) **do**
8: **if** ($\overrightarrow {r_{3}} < 0.5$) **then**
9: **if** ($|\overrightarrow {A}| < 1$) **then**
10: **Update** current search agent position using Eq. 1
11: **else**
12: **Select** a random search agent $\overrightarrow {G}_{rand}$
13: **Update** current search agent position by Eq. 4
14: **end if**
15: **else**
16: **Update** current search agent position by Eq. 2
17: **end if**
18: **end for**
19: **Update** $\overrightarrow {a}$, $\overrightarrow {A}$, $\overrightarrow {C}$, $l$, $\overrightarrow {r_{3}}$
20: **Calculate** fitness function $F_{n}$ for each $\overrightarrow {G}_{i}$
21: **Find** best individual $\overrightarrow {G^{*}}$
22: **Set** t = t +1. (increase counter).
23: **end while**
24: return $\overrightarrow {G^{*}}$

### E. Stochastic Fractal Search

**FIGURE 2.**
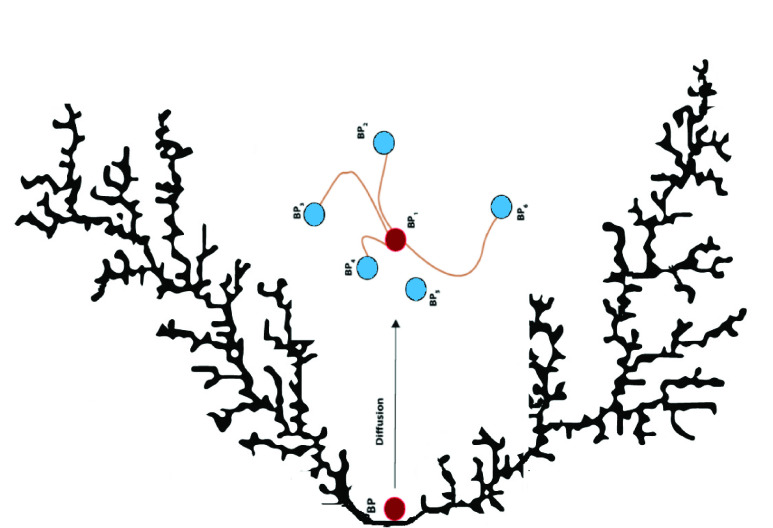
SFS algorithm; Random fractal sample with diffusion around the best solution.

The Stochastic Fractal Search (SFS) technique was proposed by [56] in which the fractal mathematical concept was used as a property of objects’ self-similarity. The Fractal Search (FS) algorithm depending on the Diffusion Limited Aggregation (DLA)that generates the objects’ fractal-shaped. Figure 2 presents a random fractal sample. The SFS technique uses diffusion and two kinds of updating processes to outperform the original FS technique. Figure 2 shows the diffusion process of the SFS technique in a graphical form for a solution. For the best solution $BP$, a list of solutions $BP_{1}, BP_{2}, BP_{3}, BP_{4}$, and $BP_{5}$ can be listed around this best solution [57].

### F. Particle Swarm Optimization

PSO algorithm is based on the swarming pattern of flocks in nature [58], [59]. PSO algorithm simulates an animal’s social behavior such as birds. The swarms searching for food by changing their positions according to the updated velocity. PSO has several particles and each particle has the following parameters:

• Position ($x^{i} \in R^{n}$), which indicated a point in $R^{n}$ search space. The fitness function is used to evaluate the particles’ current positions.
• Velocity or rate of position change, ($v^{i}$),
• Last best positions ($p^{i}$), which store better positions’ values of the particles.

During the algorithm iterations, the positions and velocity of all particles are changing. The particles’ positions are updated as follows:

\begin{equation*} x^{i}_{(t+1)}= x^{i}_{(t)} + v^{i}_{(t+1)}\tag{5}\end{equation*}

where $x^{i}_{t+1}$ is the new particle position, and the updated velocity of each particle $v^{i}_{t+1}$ can be calculated as

\begin{equation*} v^{i}_{(t+1)} = \omega v^{i}_{(t)} + C_{1} r_{1} (p^{i}_{(}t)- x^{i}_{(t)}) + C_{2} r_{2} (G - x^{i}_{(t)})\tag{6}\end{equation*}

where $\omega $ is the inertia weight, $C_{1}$ and $C_{2}$ represent cognition learning factor and the social learning factor. Parameter $G$ is the global best position and $r_{1}$ and $r_{2}$ are random numbers in [0; 1].

### G. Classification Methods

SVM can perform classification, regression, and outlier detection [21]. SVMs are suited for the classification of complex datasets. The classification of the SVM technique is based on transforming the features dimension space that is nonlinearly separable into a higher dimension space in which a hyperplane can easily separate the different classes. That can be done using a kernel trick in which linear, polynomial, or Gaussian RBF kernel can be used to decrease the computational complexity associated with the calculations of added features. The margin between classes depends on dataset instances called support vectors. While the kernel hyperparameters are those parameters that determine the margin of separation between classes and the tolerance for permitting margin violation. Even though SVM is a binary classifier, it can be easily extended to be used in multiclass classification.

KNN method can also be used for classification and regression [23] purposes. As a classifier, this algorithm considers $k$ closest training examples in the feature space. The output in this algorithm is a class membership. DT [24] is also a machine learning capable of doing both classification and regression.

MLP is a class of feedforward ANN [22]. There are three layers in MLP: input, hidden, and output layers. Such architecture with three layers is mostly suited to small or medium datasets. In addition, the dataset complexity can be accommodated using suitable activation functions and/or a suitable number of perceptrons in the hidden layers. However, large datasets can be more complex to be accommodated by only three layers of nodes. Therefore, architectures with more than three layers are common while suitable training techniques for them are usually called deep learning. That architecture can capture the complex relations associated with the large dataset they try to model or classify. The problem might arise when a small dataset with a large number of attributes needs to be used in MLP of complex architectures of many layers.

### H. Ensemble Learning

Ensemble Learning is the aggregation of a group of predictors (such as classifiers), which can often achieve better predictions. It is recommended to use diverse, independent classifiers in such methods to get the best outcome [60]. One way to achieve this is to use different learning algorithms.

To create a better classifier, the predictions of each classifier can be aggregated and then determine the class with the most votes. This is called the majority-vote classifier which is considered a hard-voting classifier. Using this approach will raise the chance that the individual classifiers will make very different types of errors to improve the ensemble’s accuracy. Another way is to use the same algorithms with different data subsets such as the Random forest. In that ensemble classifier, “forest” is an analogy that refers to creating decision trees that is trained by “bagging” method.

In bagging, a similar learning algorithm is used for all the predictors. To get the most reliable income, however, it is recommended to train them on different random subsets of the training set while sampling is performed with replacement. The general idea of this method is to increase the overall result accuracy due to the soft-computing nature of all methods in this area. Another type of ensemble classification is AdaBoost [61] in which the output of the weak learners, other learning algorithms, is collected into a weighted sum and this represents the boosted classifier final output.

## IV. Proposed Framework

The proposed framework has three phases. The first phase has a feature engineering process which includes the CNN training techniques. The second phase represents the proposed SFS-Guided WOA for feature selection and then applying the LSH-SMOTE method for balancing the selected features. The last phase, phase three, applies the proposed voting classifier algorithm (PSO-Guided WOA) for the selected features from the second phase to classify the infected cases.

### A. First Phase

In the first phase of the proposed framework, CNN is used. As dsicussed above, CNN reduce the parameters of a given problem using spatial relationships between them, which makes them a more practical classifier specially in image processing where we deal with a large number of parameters (pixels), rotation, translation, and scale of images.

Several CNN models including AlexNet [14], VGG-Net (VGG16Net and VGG19Net) [15], GoogLeNet [16], and ResNet-50 [17] are involved in this phase as shown in Fig. 4. In the CNN models, classification accuracy correlates with the extended number of convolution layers. The pre-trained CNN models are employed in this phase.

**FIGURE 3.**
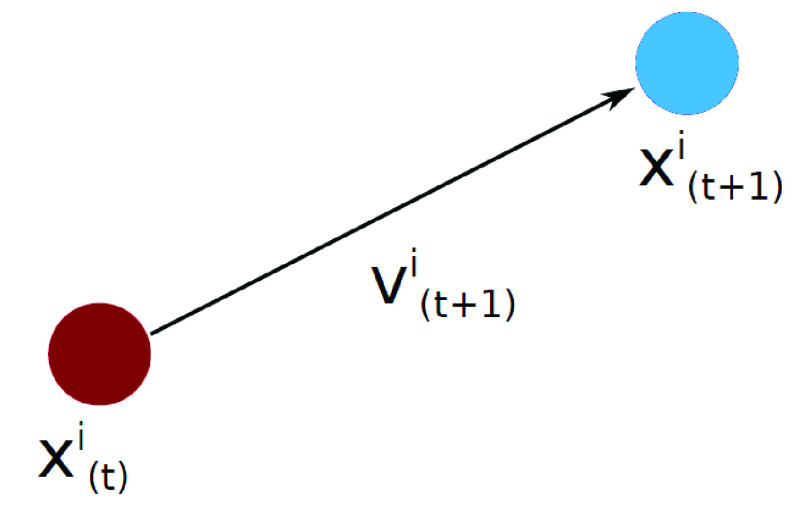
How to move a particle in the PSO algorithm.

**FIGURE 4.**
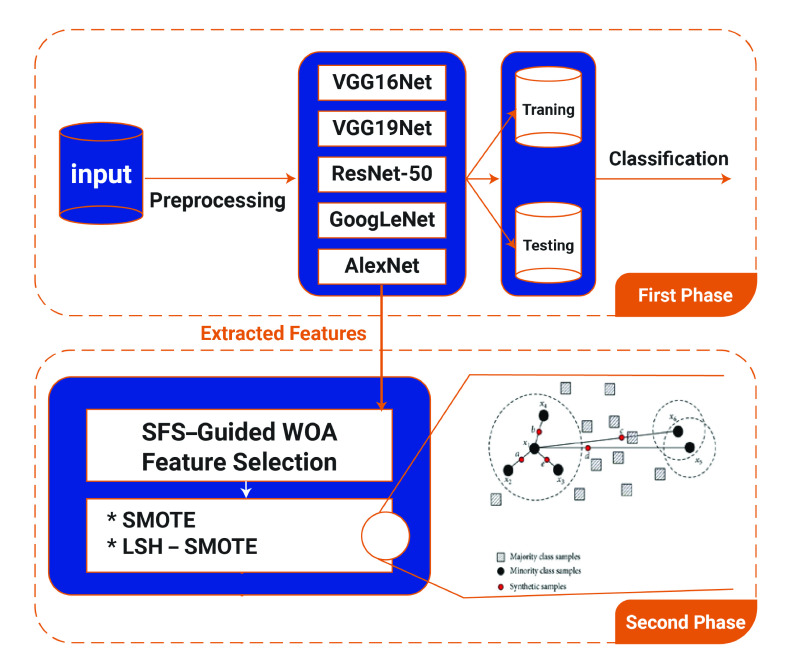
First and second phases of the proposed framework for COVID-19 patient classification.

To understand the CT images in the datasets, a Radiology Registrar at the Typical Medical complex in Riyadh and a Fellow of The Royal College of Radiologists in UK help the authors. They guided the authors to deal with COVID-19 CT images of the infected cases to differentiate them from the non-infected cases. The preprocessing step makes the data ready for the machine learning models. Based on the problem of COVID-19 and the available dataset, some data processing tasks are required before feeding the images to the learning model.

To feed the current dataset of images to the convolutional network, they must be resized to have the same size. All the CT images have been resized to $224 \times 224$ by the Nearest Neighbour interpolation function which is a simple and commonly used. The learning model can be applied in this stage for salient features extraction from CT images by altering the nodes in the fully connected layer and performing a fine-tuning using the input dataset. Then, the Min-Max-Scalar is employed for the $i$th input image $I_{i}$ normalization to be within [0, 1] by applying the following form

\begin{equation*} I'_{i}=\dfrac {I_{i} - min(I_{i})}{max(I_{i}) - min(I_{i})}\tag{7}\end{equation*}

where $I'_{i}$ is the resized image.

The data augmentation technique is applied in this research on the existing data to create new training data artificially. Image augmentation, as a type of data augmentation, creates versions of the images in the training dataset. Image transformations include horizontal and vertical shift, horizontal and vertical flip, random rotation, and random zoom are applied to the input dataset. The shift augmentation moves all pixels of the CT image in horizontal or vertical direction and keeps the image at the same dimensions. The flip process reverses all pixels rows and columns for a horizontal flip or vertical flip. The rotation augmentation rotates the CT image randomly clockwise from 0 to 360 degrees. Finally, the zoom augmentation zooms the CT image randomly by a factor range [0.9, 1.1]. The image augmentation algorithm is shown in (Algorithm 2).

Algorithm 2 **Image Augmentation Algorithm**
1: **Input** Resized CT images $\{I'\}_{i=1}^{N}$, where $N$ is the number of images and $i^{th}$ input image is denoted as $I'_{i}$
2: **Initialize** $Y_{r}$ random [0:360] and $Y_{z}$ random [0.9:1.1]
3: **for** ($i = 1: i < N + 1$) **do**
4: **Get** $I_{i}^{Vshift} =$ Vshift ($I'_{i}$)
5: **Get** $I_{i}^{Hshift} =$ Hshift ($I'_{i}$)
6: **Get** $I_{i}^{Vflip} =$ Vflip ($I'_{i}$)
7: **Get** $I_{i}^{Hflip} =$ Hflip ($I'_{i}$)
8: **Get** $I_{i}^{Rot} =$ Rotation ($I'_{i},Y_{r}$)
9: **Get** $I_{i}^{Zoom} =$ Zoom ($I'_{i},Y_{z}$)
10: **end for**
11: **Output** $I_{i}^{Vshift}, I_{i}^{Hshift}, I_{i}^{Vflip}, I_{i}^{Hflip}, I_{i}^{Rot}, I_{i}^{Zoom}$, (Image transformations)

### B. Second Phase

One of the most powerful methods to solve applications in radiology problems are Meta-heuristic algorithms. Optimization is the process by which the best possible solution is found for a particular problem from all the available solutions. The acceptable solutions are provided by these optimization techniques with less computational effort in a reasonable time. This section describes the proposed (SFS-Guided WOA) algorithm for feature selection. The numerical features that are extracted from the first phase of the CNN model are the input to the second phase for the proposed algorithm as shown in Fig. 4. The SMOTE and LSH-SMOTE methods are then applied for balancing the selected features for improving the accuracy of COVID-19 classification at the last phase.

#### 1) Guided WOA

The Guided WOA is a modified version of the original WOA. To overcome the drawback of this method, the search strategy for one random whale can be replaced with an advanced strategy that can move the whales rapidly toward the best solution or prey. From the original WOA, Eq. 4 forces whales to move around each other randomly which is similar to the global search. In the modified WOA (Guided WOA), to enhance exploration performance, a whale can follow three random whales instead of one. This can force whales for more exploration and not being affected by the leader position by replacing Eq. 4 with the following equation

\begin{align*} \overrightarrow {G}{(t+1)}=&\overrightarrow {w_{1}} * \overrightarrow {G}_{rand1} \\&+ \overrightarrow {z} * \overrightarrow {w_{2}} * (\overrightarrow {G}_{rand2} - \overrightarrow {G}_{rand3}) \\&+ (1-\overrightarrow {z}) * \overrightarrow {w_{3}} * (\overrightarrow {G} - \overrightarrow {G}_{rand1})\tag{8}\end{align*}

where $\overrightarrow {G}_{rand1}$, $\overrightarrow {G}_{rand2}$, and $\overrightarrow {G}_{rand3}$ are three random solutions. $\overrightarrow {w_{1}}$ is random value between [0, 0.5]. $\overrightarrow {w_{2}}$ and $\overrightarrow {w_{3}}$ are two random values between [0, 1]. $\overrightarrow {z}$ decreases exponentially instead of linearly to smoothly change between exploitation and exploration and calculated as

\begin{equation*} \overrightarrow {z}=1-\left ({\frac {t}{Max_{iter}}}\right)^{2}\tag{9}\end{equation*}

where $t$ represents iteration number and $Max_{iter}$ indicates maximum number of iterations. The proposed SFS-Guided WOA algorithm is shown in (Algorithm 3).

Algorithm 3 **Pseudo-Code of Proposed SFS-Guided WOA**
1: **Initialize** WOA population $\overrightarrow {G}_{i} (i = 1, 2, \ldots, n)$ with size $n$, maximum iterations $Max_{iter}$, fitness function $F_{n}$.
2: **Initialize** WOA parameters $\overrightarrow {a}$, $\overrightarrow {A}$, $\overrightarrow {C}$, $l$, $\overrightarrow {r_{1}}$, $\overrightarrow {r_{2}}$, $\overrightarrow {r_{3}}$
3: **Initialize** Guided WOA parameters $\overrightarrow {w_{1}}$, $\overrightarrow {w_{2}}$, $\overrightarrow {w_{3}}$
4: **Set** t = 1
5: **Convert** solution to binary [0 or 1].
6: **Calculate** fitness function $F_{n}$ for each $\overrightarrow {G}_{i}$
7: **Find** best individual $\overrightarrow {G^{*}}$
8: **while** $t \leq Max_{iter}$ (Termination condition) **do**
9: **for** ($i = 1: i < n + 1$) **do**
10: **if** ($\overrightarrow {r_{3}} < 0.5$) **then**
11: **if** ($|\overrightarrow {A}| < 1$) **then**
12: **Update** position of current search agent as $\overrightarrow {G}{(t+1)}= \overrightarrow {G}^{*}(t)- \overrightarrow {A}. \overrightarrow {D}$
13: **else**
14: **Select** three random search agents $\overrightarrow {G}_{rand1}$, $\overrightarrow {G}_{rand2}$, and $\overrightarrow {G}_{rand3}$
15: **Update** ($\overrightarrow {z}$) by the exponential form of $\overrightarrow {z}=1-\left ({\frac {t}{Max_{iter}}}\right)^{2}$
16: **Update** position of current search agent as $\overrightarrow {G}{(t+1)} = \overrightarrow {w_{1}} * \overrightarrow {G}_{rand1} + \overrightarrow {z} * \overrightarrow {w_{2}} * (\overrightarrow {G}_{rand2} - \overrightarrow {G}_{rand3}) + (1-\overrightarrow {z}) * \overrightarrow {w_{3}} * (\overrightarrow {G} - \overrightarrow {G}_{rand1})$
17: **end if**
18: **else**
19: **Update** position of current search agent as $\overrightarrow {G}(t+1)= \overrightarrow {D}'.e^{bl}.cos(2\pi l) + \overrightarrow {G}^{*}(t)$
20: **end if**
21: **end for**
22: **for** ($i = 1: i < n + 1$) **do**
23: Calculate $\overrightarrow {G'^{*}_{i}} = Gaussian (\mu _{\overrightarrow {G^{*}}},\sigma) + (\eta \times \overrightarrow {G^{*}} - \eta ' \times \overrightarrow {P_{i}})$
24: **end for**
25: **Update** $\overrightarrow {a}$, $\overrightarrow {A}$, $\overrightarrow {C}$, $l$, $\overrightarrow {r_{3}}$
26: **Convert** updated solution to binary by Eq. 11.
27: **Calculate** fitness function $F_{n}$ for each $\overrightarrow {G}_{i}$
28: **Find** best individual $\overrightarrow {G^{*}}$
29: **Set** t = t +1
30: **end while**
31: return $\overrightarrow {G^{*}}$

#### 2) Diffusion Procedure of SFS

Based on the diffusion procedure of the SFS algorithm, a series of random walks around the best solution can be created. This increases the exploration capability of the Guided WOA using this diffusion process for getting the best solution. The Gaussian random walks as a part of the diffusion process around the updated best position $\overrightarrow {G^{*}}$ is calculated as

\begin{equation*} \overrightarrow {G'^{*}_{i}} = Gaussian (\mu _{\overrightarrow {G^{*}}},\sigma) + (\eta \times \overrightarrow {G^{*}} - \eta ' \times \overrightarrow {P_{i}})\tag{10}\end{equation*}

where $\overrightarrow {G'^{*}_{i}}$ is the updated best solution based on the diffusion process. The parameters of $\eta $ and $\eta '$ are random numbers in [0, 1]. $\overrightarrow {G^{*}}$ and $\overrightarrow {P_{i}}$ indicate the best point position and the $i$th point in the surrounding group. $\mu _{\overrightarrow {G^{*}}}$ is equal to $\left |{ \overrightarrow {G^{*}} }\right | $ and $\sigma $ is equal to $\left |{ \overrightarrow {P_{i}} - \overrightarrow {G^{*}} }\right | $ since the number of generation around the best solution decreases.

#### 3) Binary Optimizer

For the feature selection, the solution is converted to a binary solution of 0 or 1. The following sigmoid function is applied to convert the continues solution to a binary one

\begin{align*} \overrightarrow {G}_{d}^{(t+1)}=&\begin{cases} 1 & if Sigmoid(G_{Best}) \geq 0.5 \\ 0 & otherwise \end{cases}, \\ Sigmoid(G_{Best})=&\dfrac {1}{1+\exp ^{-10(G_{Best}-0.5)}}\tag{11}\end{align*}

where $G_{Best}$ is the best position at iteration $t$. The role of the $Sigmoid$ function is to scale the continuous values between 0 and 1. The condition of $Sigmoid(G_{Best}) \geq 0.5$ is used to decide whether the value of the dimension will be 0 or 1.

#### 4) Selected Features Balance

The LSH-SMOTE technique is employed in this research to balance the selected features by the proposed SFS-Guided WOA algorithm to improve the performance of the classification algorithm. The LSH-SMOTE technique consists of the following steps:

1) LSH-SMOTE initialization,
2) converting the minority class instances into vectors,
3) creating Hash Codes by using Hash Functions then creating Hash Tables,
4) creating the nearest Neighbors List,
5) Synthetic instances generation using the SMOTE algorithm.

#### 5) Computational Complexity Analysis

The SFS-Guided WOA algorithm’ computational complexity according to Algorithm (3) will be discussed. Let $n$ as number of population; $M_{t}$ as total number of iterations. For each part of the algorithm, the time complexity can be defined as:

• Population initialization: $O$ (1).
• Parameters initialization: $\overrightarrow {a}$, $\overrightarrow {A}$, $\overrightarrow {C}$, $l$, $\overrightarrow {r_{1}}$, $\overrightarrow {r_{2}}$, $\overrightarrow {r_{3}}$, $\overrightarrow {w_{1}}$, $\overrightarrow {w_{2}}$, $\overrightarrow {w_{3}}$: $O$ (1).
• Iteration counter initialization: $O$ (1).
• Binary conversion: $O$ ($n$).
• Objective function evaluation: $O$ ($n$).
• Finding the best individual: $O$ ($n$).
• Position updating: $O$ ($M_{t} \times n$).
• Diffusion process calculation: $O$ ($M_{t} \times n$).
• Updating $\overrightarrow {a}$ by the exponential form: $O$ ($M_{t}$).
• Updating parameters $\overrightarrow {a}$, $\overrightarrow {A}$, $\overrightarrow {C}$, $l$, $\overrightarrow {r_{3}}$: $O$ ($M_{t}$).
• Converting updated solution to binary: $O$ ($M_{t} \times n$).
• Objective function evaluation: $O$ ($M_{t} \times n$).
• Best individual update: $O$ ($M_{t} \times n$).
• Iteration counter increment: $O$ ($M_{t}$).

As per the above complexities, the overall complexity of the proposed SFS-Guided WOA algorithm is $O$ ($M_{t} \times n$). Considering the number of variables as $m$, the final computational complexity of the algorithm will be $O$ ($M_{t} \times n \times m$).

### C. Third Phase

**FIGURE 5.**
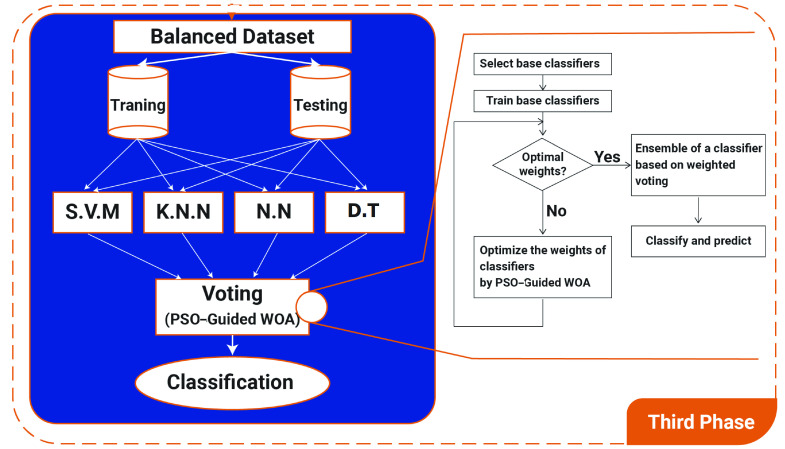
Third phase of the proposed framework for COVID-19 patient classification.

The third and last phase is the classification of infected patients. Figure 5 shows the third phase of the proposed framework for COVID-19 patient classification. In this section, a voting classifier is proposed based on PSO and Guided WOA algorithms as shown in Algorithm 4. The PSO-Guided WOA aggregates the SVM, NN, KNN, and DT classifiers to improve the ensemble’s accuracy. After balancing the selected features by the SMOTE or LSH-SMOTE algorithms, the classifiers are trained to get the optimal weights. The PSO-Guided WOA starts to optimize theses weights.

Algorithm 4 **Pseudo-Code of Proposed PSO-Guided WOA**
1: **Initialize** WOA population $\overrightarrow {G}_{i} (i = 1, 2, \ldots, n)$ with size $n$, maximum iterations $Max_{iter}$, fitness function $F_{n}$.
2: **Initialize** WOA parameters $\overrightarrow {a}$, $\overrightarrow {A}$, $\overrightarrow {C}$, $l$, $\overrightarrow {r_{1}}$, $\overrightarrow {r_{2}}$, $\overrightarrow {r_{3}}$
3: **Initialize** Guided WOA parameters $\overrightarrow {w_{1}}$, $\overrightarrow {w_{2}}$, $\overrightarrow {w_{3}}$
4: **Set** t = 1
5: **Calculate** fitness function $F_{n}$ for each $\overrightarrow {G}_{i}$
6: **Find** best individual $\overrightarrow {G^{*}}$
7: **while** $t \leq Max_{iter}$ (Termination condition) **do**
8: **if** ($t \% 2 == 0$) **then**
9: **for** ($i = 1: i < n + 1$) **do**
10: **if** ($\overrightarrow {r_{3}} < 0.5$) **then**
11: **if** ($|\overrightarrow {A}| < 1$) **then**
12: **Update** position of current search agent as $\overrightarrow {G}{(t+1)}= \overrightarrow {G}^{*}(t)- \overrightarrow {A}. \overrightarrow {D}$
13: **else**
14: **Select** three random search agents $\overrightarrow {G}_{rand1}$, $\overrightarrow {G}_{rand2}$, and $\overrightarrow {G}_{rand3}$
15: **Update** ($\overrightarrow {z}$) by the exponential form of
16: $\overrightarrow {z}=1-\left ({\frac {t}{Max_{iter}}}\right)^{2}$ Update position of current search agent as $\overrightarrow {G}{(t+1)} =\vphantom {_{\int }} \overrightarrow {w_{1}} * \overrightarrow {G}_{rand1} + \overrightarrow {z} * \overrightarrow {w_{2}} * (\overrightarrow {G}_{rand2} - \overrightarrow {G}_{rand3}) + (1-\overrightarrow {z}) * \overrightarrow {w_{3}} * (\overrightarrow {G} - \overrightarrow {G}_{rand1})$
17: **end if**
18: **else**
19: **Update** position of current search agent as $\overrightarrow {G}(t+1)= \overrightarrow {D}'.e^{bl}.cos(2\pi l) + \overrightarrow {G}^{*}(t)$
20: **end if**
21: **end for**
22: **Calculate** fitness function $F_{n}$ for each $\overrightarrow {G}_{i}$ from Guided WOA
23: **else**
24: Calculate fitness function $F_{n}$ for each $\overrightarrow {G}_{i}$ from PSO
25: **end if**
26: **Update** $\overrightarrow {a}$, $\overrightarrow {A}$, $\overrightarrow {C}$, $l$, $\overrightarrow {r_{3}}$
27: **Find** best individual $\overrightarrow {G^{*}}$
28: **Set** t = t +1
29: **end while**
30: return $\overrightarrow {G^{*}}$

For the proposed Algorithm 4, the guided WOA in section IV-B1 is employed in the algorithm development. After the initialization of the WOA algorithm and find the first best solution $\overrightarrow {G^{*}}$ (Lines from 1 to 6), the iteration number $t$ starts to divide the calculation of the fitness function from the guided WOA or from the PSO algorithm. If $t \% 2 == 0$ (Line 8), then the algorithm goes through the updating positions and calculating the fitness function $F_{n}$ for the updated solutions from the guided WOA (Lines from 9 to 22). Otherwise, the fitness function $F_{n}$ will be calculated based on The PSO algorithm (Line 24).

#### 1) Computational Complexity Analysis

The proposed PSO-Guided WOA algorithm’ computational complexity will be discussed here according to Algorithm (4).Let $n$ as number of population; $M_{t}$ as number of iterations. For each part of the algorithm, the time complexity can be defined as:

• Population initialization: $O$ (1).
• Parameters initialization $\overrightarrow {a}$, $\overrightarrow {A}$, $\overrightarrow {C}$, $l$, $\overrightarrow {r_{1}}$, $\overrightarrow {r_{2}}$, $\overrightarrow {r_{3}}$, $\overrightarrow {w_{1}}$, $\overrightarrow {w_{2}}$, $\overrightarrow {w_{3}}$: $O$ (1).
• Iteration counter initialization: $O$ (1).
• Objective function evaluation: $O$ ($n$).
• Determining the best solution: $O$ ($n$).
• Position updating: $O$ ($M_{t} \times n$).
• Objective function evaluation for each individual from Guided WOA: $O$ ($M_{t} \times n$).
• Fitness function calculation for each individual from PSO: $O$ ($M_{t} \times n$).
• Updating parameters $\overrightarrow {a}$, $\overrightarrow {A}$, $\overrightarrow {C}$, $l$, $\overrightarrow {r_{3}}$: $O$ ($M_{t}$).
• Best solution update: $O$ ($M_{t} \times n$).
• Iteration counter increament: $O$ ($M_{t}$).

Thus, the overal complexity PSO-Guided WOA algorithm is $O$ ($M_{t} \times n$). Considering a problem with $m$ variables, the final computational complexity of the algorithm will be $O$ ($M_{t} \times n \times m$).

### D. Objective Function

Objective functions are used to evaluate the solutions in an optimization algorithm. The function is depending on two parameters of the classification error rate and the number of selected features. The solution is good if the subset of features gives a lower number of selected features and a lower classification error rate. The following equation is used to get the quality of each solution

\begin{equation*} F_{n} = h_{1} E(D)+ h_{2} \dfrac {|s|}{| f|}\tag{12}\end{equation*}

where $E(D)$ is the rate of error for the optimizer, $s$ indicates the number of selected features, $f$ indicates the total number of features and $h_{1} \in [{0,1}], h_{2}= 1- h_{1}$ manage the importance of the number of the selected feature for population with size $n$ and the classification error rate.

## V. Experimental Results

The experiments section in this article is divided into three scenarios. The first scenario is based on the first phase of the proposed model. This experiment shows the effectiveness of different CNN models for classifying the COVID-19 cases and interns show the importance of extracting features for the next phase. In the second scenario, the proposed feature selection algorithm (SFS-Guided WOA) is tested and compared to other algorithms to show its performance. The third scenario is designed to investigate the ability of the proposed voting optimizer (PSO-Guided WOA) for improving the classification accuracy of the COVID-19 cases. Finally, Wilcoxon’s rank-sum test and t-test are performed to verify the superiority of the proposed algorithms in a statistical way. The CT images datasets, [49], are separated randomly in the experiment of the first scenario into (60%, 20%, 20%) images for the training, validation, and testing processes.

### A. First Scenario: Model’s First Phase

The first experiment is designed to investigate the classification accuracy of five CNN models namely AlexNet [14], VGG-Net (VGG16Net and VGG19Net) [15], GoogLeNet [16], and ResNet-50 [17] for the tested dataset. In this scenario, several performance metrics are calculated to measure the performance of the different models for COVID-19 classification. Table 3 shows the CNN experimental setup employed in the first scenario. The default parameters are employed in this experiment since the first stage is used to extract features of the CT images from the earlier layers of a CNN model to be used for the next scenario for features selection and balancing.

**TABLE 3:** CNN Experimental Setup

Parameter	Value
CNN Default training options	
Momentum Learn	0.9000
RateDropFactor	0.1000
LearnRateDropPeriod	10
L2Regularization	1.0000c-04
GradientThresholdMethod	12norm
GradientThreshold	Inf
VerboseFrequency	50
ValidationData	[imds]
ValidationFrequency	50
ValidationPatience	Inf
ResetInputNormalization	1
CNN Custom training options	
\\ExecutionEnvironment	gpu
InitiallearnRate	1.0000e-04
MaxEpochs	20
MiniBatchSize	8
Shuffle	every-epoch
Verbose	0
Optimizer	sgdm
LearnRateSchedule	piecwise

#### 1) First Scenario: Performance Metrics

The performance metrics calculated for the first phase are accuracy, sensitivity, specificity, precision (PPV), Negative Predictive Value (NPV), and F-score. Let $TP$ represents the true-positive value and $TN$ represents the true-negative value, while $FN$ indicates the false-negative value and $FP$ indicates the false-positive value. The metrics are defined as in the following equations.

• **Accuracy:** measures the model ability to identify the whole cases correctly, regardless the cases are being positive or negative and can be formed as
  \begin{equation*} Accuracy = \dfrac {TP + TN}{TP + TN + FP + FN}\tag{13}\end{equation*}
• **Sensitivity:** called the true positive rate (TPR) or recall. It computes the capability of the positive case and is calculated as
  \begin{equation*} Sensitivity = \dfrac {TP}{TP + FN}\tag{14}\end{equation*}
• **Specificity:** called the true negative rate (TNR) or selectivity. It gets the capability of finding negative cases and is calculated as
  \begin{equation*} Specificity = \dfrac {TN}{TN + FP}\tag{15}\end{equation*}
• **Precision:** called positive predictive value (PPV). It directs the rate of true positives among all positive values. It is calculated as
  \begin{equation*} PPV = \dfrac {TP}{TP + FP}\tag{16}\end{equation*}
• **Negative Predictive Value (NPV):** It directs rate of true negatives among all negative values. It is calculated as
  \begin{equation*} VPV = \dfrac {TN}{TN + FN}\tag{17}\end{equation*}
• **F-score:** measures the harmonic mean of precision and sensitivity and is calculated as
  \begin{equation*} F-score = 2 \times \dfrac {PPV \times TPR}{PPV + TPR}\tag{18}\end{equation*}

#### 2) First Scenario: Results and Discussion

This scenario results are shown in Table 4. The results show that the precision (Pvalue) of the GoogLeNet model of 84.75% which is better than VGG19Net (83.78%), ResNet-50 (81.08%), AlexNet (75%), and VGG16Net (51.75%) models. The AlexNet model outperforms other models with an F-score of 77.88%. However, the GoogLeNet model has better specificity of 92.44% than other models. According to sensitivity, the rate of the VGG16Net model of 95.08% is better than the sensitivity rate of AlexNet (81%), ResNet-50 (62.5%), VGG19Net (62%), and GoogLeNet (50%) models, respectively. For the Pvalue, the VGG16Net model has a better percentage of 87.74%. As an overall performance metric for the models, the AlexNet model has an accuracy of 79% whereas VGG19Net, ResNet-50, GoogLeNet, and VGG16Net have the accuracy of 77.17%, 77.17%, 73.06%, and 58.21% for the tested COVID-19 dataset, respectively.

**TABLE 4:** Comparison of the Performance Metrics for the COVID-19 Classification Based on CNN Models

CNN Models/Metric	Accuracy	Sensitivity (TPR)	Specificity (TNR)	Pvalue (PPV)	Nvalue (NPV)	F-score
AlexNet	0.7900	0.8100	0.7731	0.7500	0.8288	0.7788
VGG16Net	0.5821	0.9508	0.2844	0.5175	0.8774	0.6702
VGG19Net	0.7717	0.6200	0.8992	0.8378	0.7379	0.7126
GoogLeNet	0.7306	0.5000	0.9244	0.8475	0.6875	0.6289
ResNet-50	0.7717	0.6250	0.8862	0.8108	0.7517	0.7059

Based on this experiment, the highest accuracy that can be achieved for the CT images from the COVID-19 dataset tested in this research is 79% by the AlexNet model. Since this is not acceptable accuracy in this critical endeavor, the features are extracted from the earlier layers of the AlexNet model, according to its promising performance, to be used for the next scenario for features selection and balancing.

### B. Second Scenario: Model’s Second Phase

In this scenario, the importance and performance of the proposed feature selection algorithm (SFS-Guided WOA) are investigated. The proposed algorithm in the second phase is compared to other algorithms of the original WOA [25], Grey Wolf Optimizer (GWO) [26], Genetic Algorithm (GA) [27], PSO [28], hybrid of PSO and GWO (GWO-PSO) [29], hybrid of GA and GWO (GWO-GA), Bat Algorithm (BA) [30], Biogeography-Based Optimizer (BBO) [31], Multiverse Optimization (MVO) [32], Bowerbird Optimizer (SBO) [33], and Firefly Algorithm (FA) [34] in terms of average error, average select size, average (mean) fitness, best fitness, worst fitness, and standard deviation fitness, to show its performance. Table 5 shows the configuration of the proposed (SFS-Guided WOA) algorithm in the experiments. The parameters of $h_{1}$ and $h_{2}$ in the fitness function are assigned to 0.99 and 0.01, respectively. Table 6 shows the configuration of the compared algorithms in the experiments.

**TABLE 5:** Proposed (SFS-Guided WOA) Algorithm Configuration

Parameter	Value
Number of search agents	10
Number of iterations	80
Number repetitions of runs	20
Problem dimension	Number of features
Search domain	[0,1]
K-neighbors	5
K-fold cross-validation	10
Maximum diffusion level	1
$h_{1}$ Parameter in $F_{n}$	0.99
$h_{2}$ Parameter in $F_{n}$	0.01

**TABLE 6:** Compared Algorithms Configuration for Feature Selection

Algorithm	Parameter (s)	Value (s)
GWO	$a$	2 to 0
PSO	Inertia $W_{max}$, $W_{min}$	[0.9, 0.6]
	Acceleration constants $C_{1}$, $C_{2}$	[2, 2]
GA	Mutation ratio	0.1
	Crossover	0.9
	Selection mechanism	Roulette wheel
SBO	Step size	0.94
	Mutation probability	0.05
	Difference between the upper and lower limit	0.02
WOA	$a$	2 to 0
	$r$	[0, 1]
MVO	Wormhole existence probability	[0.2,1]
FA	Number of fireflies	10
BA	Pluse rate	0.5
	Loudness	0.5
	Frequency	[0, 1]
BBO	Probability of Immigration	[0, 1]
	Probability of Mutation	0.05
	Probability of Habitat modification	1.0
	Step size	1.0
	Migration rate	1.0
	Maximum immigration	1.0

#### 1) Second Scenario: Performance Metrics

For the evaluation of the proposed SFS-Guided WOA algorithm effectiveness, the following metrics are employed. Let $M$ is the number repetitions of runs of an optimizer for the feature selection problem; $g_{j}^{*}$ is the best solution at the run number $j$; $N$ is the number of tested points.

• **Average Error** is calculated to show the accuracy of the classifier in giving the selected feature set. It is calculated as
  \begin{equation*} AvgError = 1- \frac {1}{M} \sum _{j=1}^{M} \frac {1}{N} \sum _{i=1}^{N} Match(C_{i}, L_{i})\tag{19}\end{equation*}
  where $C_{i}$ is the label of the classifier output for point $i$, and $L_{i}$ is the label of the class for point $i$, and $Match$ calculates the matching between two inputs.
• **Average Fitness** is the selected features average size to the total number of features in the dataset ($D$). It is calculated as
  \begin{equation*} AvgSelectSize = \frac {1}{M} \sum _{j=1}^{M} \dfrac {size(g_{j}^{*})}{D}\tag{20}\end{equation*}
  where $size(g_{j}^{*})$ is the size of the vector $g_{j}^{*}$.
• **Mean** is the average of the solutions output from running an optimizer for several times $M$. It is calculated as
  \begin{equation*} Mean = \frac {1}{M} \sum _{j=1}^{M} g_{j}^{*}\tag{21}\end{equation*}
• **Best Fitness** is the minimum fitness function of an optimizer running for several times $M$. It is calculated as
  \begin{equation*} BestF_{n} = Min_{j=1}^{M} g_{j}^{*}\tag{22}\end{equation*}
• **Worst Fitness** is the worst solution found by an optimizer running for several times $M$. It is calculated as
  \begin{equation*} WorstF_{n} = Max_{j=1}^{M} g_{j}^{*}\tag{23}\end{equation*}
• **Standard Deviation (SD)** is the obtained best solutions variation which can be found by running an optimizer several times $M$. SD can be calculated as
  \begin{equation*} SD = \sqrt {\frac {1}{M-1} \sum (g_{j}^{*} - Mean)^{2} }\tag{24}\end{equation*}
  where $Mean$ is the average defined in equation 21.

#### 2) Second Scenario: Results and Discussion

The results of the proposed SFS-Guided WOA algorithm in this experiment are shown in Table 7. The lower error indicates that the optimizer has selected the proper set of features for the next stage. The SFS-Guided WOA algorithm achieved the minimum average error of (0.1381) in selecting the proper features. The feature selection algorithms ordered from the best to the worst according to the minimum error for the current problem are SFS-Guided WOA, PSO, GWO, GWO-GA, WOA, GA, BA, GWO-PSO, FA, BBO, MVO, and lastly SBO. Note that, the proposed algorithm outperforms the original WOA algorithm. Table 7 also shows that the proposed algorithm can find the lowest fitness value (0.2013), for the selected features of the COVID-19 datasets, which is lower than the compared algorithms values. The proposed algorithm can find the best fitness value of (0.1031) compared to other optimization techniques throughout runs. On the other hand, SFS-Guided WOA can not find the worst fitness and it has the lowest standard deviation compared to other algorithms that prove the stability and robustness of the proposed algorithm.

**TABLE 7:** Performance of the Proposed Feature Selection Algorithm (SFS-Guided WOA) Compared to Other Algorithms

Metric/Optimizer	SFS-Guided WOA	GWO	GWO-PSO	PSO	BA	WOA	BBO	MVO	SBO	GWO-GA	FA	GA
Average Error	0.1381	0.1553	0.1946	0.1891	0.1987	0.1889	0.1573	0.1658	0.1974	0.1754	0.1875	0.1689
Average Select size	0.0909	0.2909	0.4242	0.2909	0.4303	0.4543	0.4547	0.3874	0.4612	0.2137	0.3254	0.2333
Average Fitness	0.2013	0.2175	0.2258	0.2159	0.2388	0.2237	0.2216	0.2456	0.2556	0.2236	0.2678	0.2289
Best Fitness	0.1031	0.1378	0.1793	0.1962	0.1285	0.1878	0.2113	0.1708	0.1987	0.2014	0.1865	0.1322
Worst Fitness	0.2016	0.2047	0.2893	0.2639	0.2301	0.2639	0.2978	0.2888	0.2784	0.2776	0.2841	0.2473
Standard Deviation Fitness	0.0236	0.0283	0.0465	0.0277	0.0376	0.0299	0.0726	0.0784	0.0886	0.0289	0.0645	0.0299

Based on this experiment, the selected features are then balanced using two methods named SMOTE and LSH-SMOTE to be ready for the classification scenario. For both algorithms, the nearest neighbors parameter $k = 5$, and the oversampling percentage is 50% of features distribution (majority class = minority class). For the SMOTE algorithm, the number of instances per leaf is equal to 2. For the LSH-SMOTE algorithm, the Hashes parameter $H = 5$ and the Hash tables parameter $T = 4$.

#### 3) Second Scenario: Wilcoxon’s Rank-Sum

For getting the p-values between the proposed SFS-Guided WOA algorithm and other algorithms, Wilcoxon’s rank-sum test is employed. This statistical test can determine if the results of the proposed algorithm and other algorithms have a significant difference or not; p-value < 0.05 will demonstrate significant superiority. By contrast, a p-value >0.05 shows that the results have no significant difference. Hypothesis testing is formulated here in terms of two hypotheses; the null hypothesis ($H_{0}$: $\mu _{SFS-Guided WOA} = \mu _{GWO}$, $\mu _{SFS-Guided WOA} = \mu _{GWO-PSO}$, $\mu _{SFS-Guided WOA} =\mu _{PSO}$, ${\dots }$, $\mu _{SFS-Guided WOA} =\mu _{GA}$) and the alternate hypothesis ($H_{1}$: Means are not all equal). Table 8 shows the results of p-value in which p-values less than 0.05 could be achieved between the proposed algorithm and other algorithms showing the superiority of the SFS-Guided WOA algorithm and indicating that the algorithm is statistically significant. Thus, the alternate hypothesis $H_{1}$ is accepted.

**TABLE 8:** p-values of SFS-Guided WOA in Comparison to Other Algorithms Using Wilcoxon’s Rank-Sum

GWO	GWO-PSO	PSO	BA	WAO	BBO	MVO	SBO	GWO-GA	FA	GA
1.13E-05	1.13E-05	1.13E-05	1.13E-05	1.13E-05	1.13E-05	1.13E-05	1.13E-05	1.13E-05	1.13E-05	1.13E-05

### C. Third Scenario: Model’s Third Phase

This scenario is divided into three experiments and statistical tests. The first experiment is designed to investigate the results for the single classifiers of SVM, KNN, NN, and DT based on balanced and unbalanced features that are selected from the second scenario. The next experiment is performed to compare the proposed voting classifier (PSO-Guided WOA) with other ensemble learning techniques. In the last experiment, the proposed algorithm is compared with other voting classifier algorithms to check its effectiveness. Statistical tests of ANOVA and T-test are performed between the compared algorithms to show the effectiveness of the proposed algorithm. Table 9 shows the configuration of the proposed (PSO-Guided WOA) algorithm in the experiments. The parameters of $h_{1}$ and $h_{2}$ in the fitness function are assigned to 0.99 and 0.01, respectively. Table 10 shows the configuration of the compared algorithms in the experiments.

**TABLE 9:** Proposed (PSO-Guided WOA) Algorithm Configuration

Parameter	Value
Number of whales	20
Number of iterations	20
Number repetitions of runs	20
Inertia $W_{max}$, $W_{min}$	[0.9, 0.6]
Acceleration constants $C_{1}$, $C_{2}$	[2, 2]
K-neighbors	5
K-fold cross-validation	10
$h_{1}$ Parameter in $F_{n}$	0.99
$h_{2}$ Parameter in $F_{n}$	0.01

**TABLE 10:** Compared Algorithms Configuration for Classification

Algorithm	Parameter (s)	Value (s)
GWO	$a$	2 to 0
	No. of wolves	20
	No. of iterations	20
PSO	Inertia $W_{max}$, $W_{min}$	[0.9, 0.6]
	Acceleration constants $C_{1}$, $C_{2}$	[2, 2]
	No. of particles	20
	Generations	20
GA	Mutation ratio	0.1
	Crossover	0.9
	Selection mechanism	Roulette wheel
	Population size	20
	Generations	20
WOA	$a$	2 to 0
	$r$	[0, 1]
	No. of whales	20
	No. of iterations	20

#### 1) Third Scenario: Performance Metrics

This scenario performance metrics are the Area Under the ROC Curve (AUC) and the Mean Square Error (MSE). AUC is a good indicator of classification performance due to being independent from the distribution of instances between classes which is also referred to as a balanced accuracy or macro-average [51]. In the current case of binary classification, the balanced accuracy is equal to the arithmetic mean of specificity and sensitivity, or AUC with binary predictions rather than scores. The AUC (balanced accuracy) value can be calculated as follows:

\begin{equation*} AUC = (Sensitivity + Specificity)/2\tag{25}\end{equation*}

The Mean Square Error (MSE) evaluates the classifiers performance, calculates the difference between the required and the actual output of the classifiers according to this equation:

\begin{equation*} MSE = \sum _{x=1}^{n} (o_{x}^{h} d_{x}^{h})^{2}\tag{26}\end{equation*}

where $n$ indicates number of outputs, $d_{x}^{h}$ indicates the $x$th input neuron optimal output when the $h$th training instance is applied, and $o_{x}^{h}$ indicates optimal output actual output of the $x$th input neuron when the $h$th training instance appears in the input.

#### 2) Third Scenario: Results and Discussion

The first experiment results for the SVM, KNN, NN, and DT as a single classifiers are shown in Table 11. The classifier results are shown based on three cases of no preprocessing, balancing selected features by the SMOTE algorithm, and balancing selected features by the LSH-SMOTE algorithm. Note from Table 11 that, the DT classifier achieved the highest AUC percentage of 0.911 with the minimum MSE of (0.007932). This result show the importance of balancing the selected features from the previous stage by the LSH-SMOTE algorithm.

**TABLE 11:** AUC and MSE of the Signal Classifiers

Metric	Preprocessing State	SVM	NN	KNN	DT
AUC	without preprocessing	0.684	0.713	0.661	0.793
	with SMOTE preprocessing	0.721	0.761	0.717	0.831
	with LSH-SMOTE preprocessing	0.847	0.867	0.836	0.911
MSE	without preprocessing	0.099845	0.082373	0.114932	0.042853
	with SMOTE preprocessing	0.089411	0.067079	0.085852	0.035723
	with LSH-SMOTE preprocessing	0.033685	0.026574	0.039543	0.007932

**FIGURE 6.**
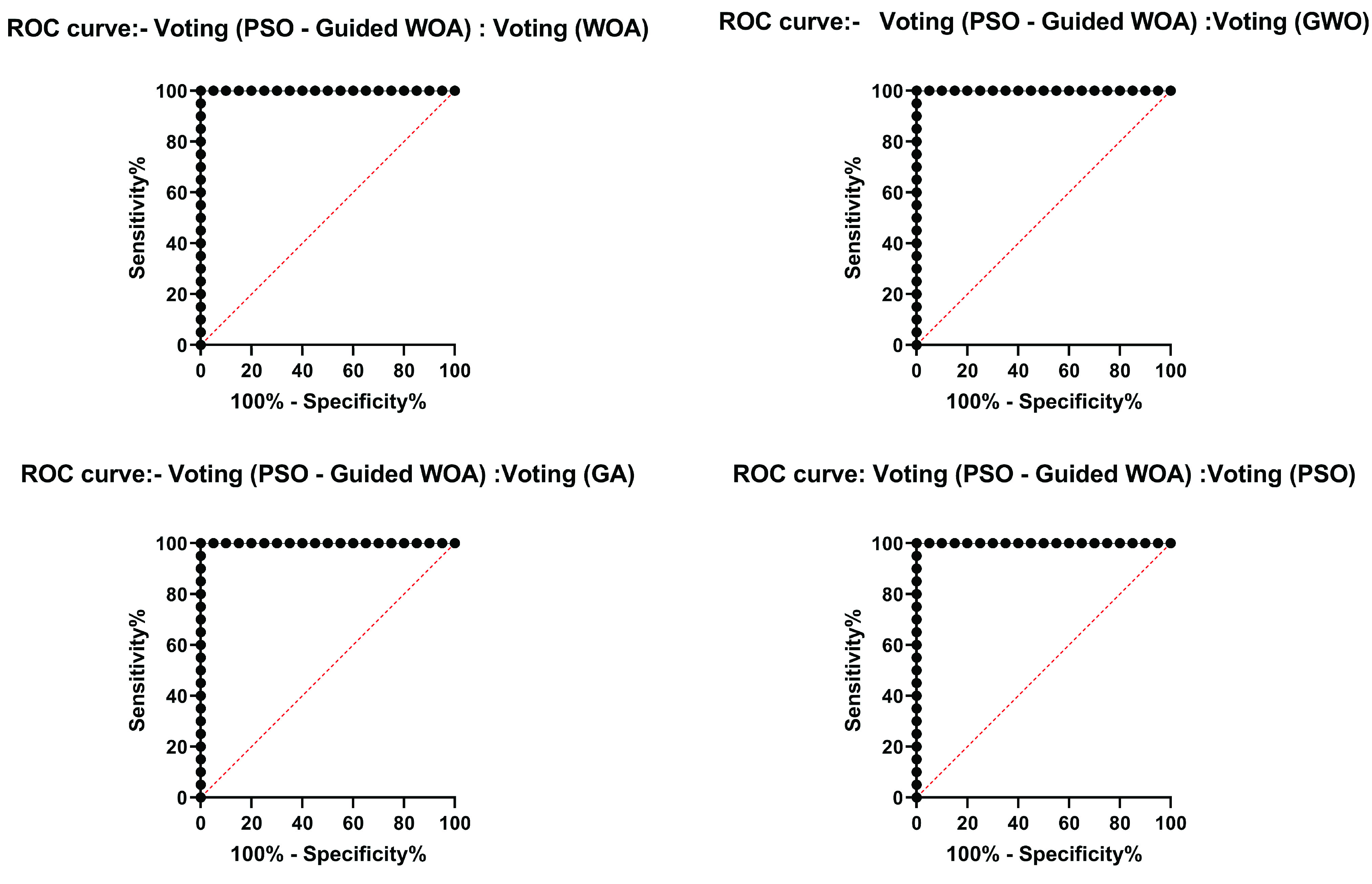
ROC curves of the proposed voting (PSO-Guided WOA) algorithm versus compared algorithms.

The next experiment results for comparing the proposed algorithm with other ensemble learning methods of Bagging, AdaBoost, and Majority voting are shown in Table 12. This table shows that the proposed voting classifier (PSO-Guided WOA) with LSH-SMOTE preprocessing can achieve AUC result of 0.995 which outperforms other ensemble learning techniques. The MSE of the proposed (2.49569E-05) is the minimum MSE compared with Bagging (0.028231), AdaBoost (0.014892), and Majority voting (0.005931) techniques. The last experiment results for comparing the voting classifier with other voting classifiers using WOA, GWO, GA, and PSO are shown in Table 13. The results show that the PSO-Guided WOA algorithm with AUC of 0.995 outperforms the voting WOA (AUC = 0.931), voting GWO (AUC = 0.946), voting GA (AUC = 0.939), and voting PSO (AUC = 0.954), respectively. Figure 6 shows the ROC curves of the proposed voting (PSO-Guided WOA) algorithm versus compared voting algorithms. These figures show that the proposed algorithm is able to distinguish between the COVID-19 and non-COVID-19 cases with a high AUC value near to 1.0 as shown in Table 13.

**TABLE 12:** Comparing the Proposed Algorithm With Other Ensemble Learning Methods

Metric/Ensemble Learning	Bagging	AdaBoost	Majority voting	Voting (PSO-Guided WOA)
AUC with LSH-SMOTE	0.842	0.877	0.924	0.995
MSE with LSH-SMOTE	0.028231	0.014892	0.005931	2.49569E-05

**TABLE 13:** Comparing the Proposed Algorithm With Other Voting Classifiers

Metric/Ensemble Learning	Voting (PSO-Guided WOA)	Voting WOA	Voting GWO	Voting GA	Voting PSO
AUC with LSH-SMOTE	0.995	0.931	0.946	0.939	0.954
MSE with LSH-SMOTE	2.49569E-05	0.006084	0.003025	0.003721	0.00151

#### 3) Third Scenario: Statistical Test

**FIGURE 7.**
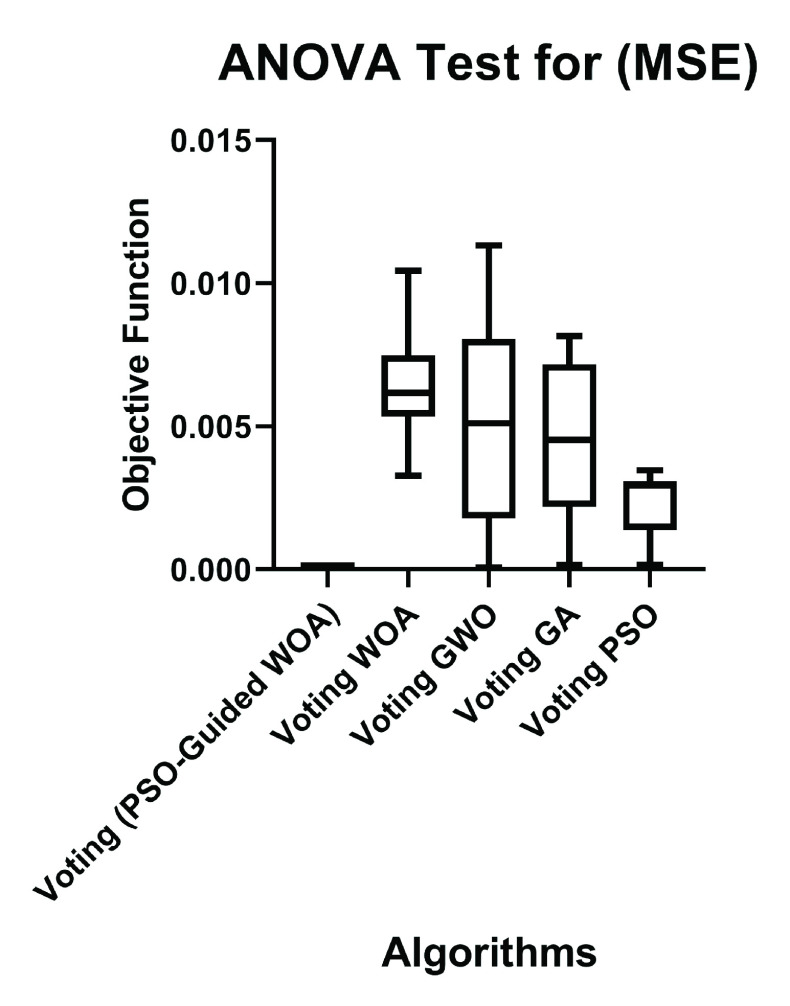
ANOVA test for different algorithms.

To conclude whether there is any statistical difference between the MSE of the proposed (PSO-Guided WOA) algorithm and other compared algorithms, a one-way analysis of variance (ANOVA) test was applied. The hypothesis testing can be formulated here in terms of two hypotheses; the null hypothesis ($H_{0}$: $\mu _{A1} = \mu _{B1} =\mu _{C1} =\mu _{D1} =\mu _{E1}$), where A1: Voting (PSO-Guided WOA), B1: Voting WOA, C1: Voting GWO, D1: Voting GA, and E1: Voting PSO, and the alternate hypothesis ($H_{1}$: Means are not all equal). The ANOVA test results are shown in Table 14. Figure 7 shows the ANOVA test for proposed and the compared algorithms versus the objective function. Based on this test results, the alternate hypothesis $H_{1}$ is accepted. However, we cannot tell which algorithm is better from ANOVA, so another test is conducted between every two algorithms.

**TABLE 14:** A One-Way Analysis of Variance (ANOVA) Test Results

Source of Variation	SS	df	MS	F	P-value	F crit
Between Groups	0.000510289	4	0.000128	25.28941	2.82E-14	2.467494
Within Groups	0.000479227	95	5.04E-06	–	–	–
Total	0.000989516	99	–	–	–	–

A one-tailed T-Test at 0.05 significance level is performed. Hypothesis testing is formulated here in terms of two hypotheses; the null hypothesis ($H_{0}$: $\mu _{A1} = \mu _{B1}, \mu _{A1} = \mu _{C1}, \mu _{A1} =\mu _{D1}, \mu _{A1} =\mu _{E1}$) and the alternate hypothesis ($H_{1}$: Means are not all equal). The results in Table 15, for 20 samples (Number repetitions of runs) as mentioned in Table 9, show that the p-values are less than 0.05 which indicates that there is a statistically significant difference between groups. Thus, the alternate hypothesis $H_{1}$ is accepted.

**TABLE 15:** A One-Tailed T-Test at 0.05 Significance Level Results. A1: Voting (PSO-Guided WOA), B1: Voting WOA, C1: Voting GWO, D1: Voting GA, and E1: Voting PSO

	A1-B1	A1-C1	A1-D1	A1-E1
Correlation	0.234821	−0.14323	0.436198	−0.3496
T.Test	2.26E-17	2.36E-07	7.65E-10	1.09E-11

## VI. Discussion

The experiments in this research are designed based on three scenarios to assess the performance and accuracy of the proposed framework for COVID-19 classification. The first scenario shows that the highest classification accuracy of the compared CNN models can be achieved by the AlexNet model for the CT images from the tested COVID-19 dataset. Based on these results, the features are extracted from the earlier layers of the AlexNet model to be used for the next scenario for features selection and balancing. In the second scenario, the performance of the proposed feature selection algorithm (SFS-Guided WOA) is assessed. Results show that the proposed algorithm outperforms the compared algorithms, including the original WOA algorithm, and could find the lowest fitness value for the feature selection of the extracted features from the COVID-19 datasets. In addition, the proposed algorithm has the lowest standard deviation compared to other algorithms that prove the stability and robustness of the proposed technique. Based on the second scenario results, the selected features are then balanced using the SMOTE and LSH-SMOTE methods to be ready for the last stage which includes the final classification. The third scenario shows the performance of the proposed classification algorithm (PSO-Guided WOA). Results show that the proposed voting classifier (PSO-Guided WOA) with LSH-SMOTE preprocessing could achieve an AUC with binary predictions (balanced accuracy) result of 0.995 and a MSE of 2.49569E-05 which outperforms other state-of-the-art ensemble learning techniques. That shows the importance of balancing the selected features from the previous stage by the LSH-SMOTE algorithm. The experimental results for comparing the voting classifier with other voting classifiers using WOA, GWO, GA, and PSO show the superiority of the proposed framework to identify COVID-19 patients using CT images. Thus, the efficacy of diagnosis can be improved while avoiding the radiologists the heavy workload associated with the initial screening of COVID-19 pneumonia.

## VII. Conclusion and Future Work

This article proposes a framework for COVID-19 classification with three cascaded phases. In the first phase, the hierarchical feature representation is automatically extracted from the training CT images by the CNN model of AlexNet. Afterward, the proposed feature selection algorithm, using SFS and Guided WOA techniques, is applied to select features in the second phase. The selected features are then balanced by the LSH-SMOTE algorithm to improve the classification results. In the last phase, a voting classifier, using PSO and Guided WOA techniques, is proposed to aggregate the predictions of four single classifiers, named SVM, NN, KNN, and DT, and predict the most voted class. This increases the chance that the individual classifiers will make very different types of errors to improve the ensemble’s accuracy. Two datasets are used to test the proposed model. The first is the COVID-19 dataset which has CT images containing clinical findings of COVID-19 and the second is the non-COVID-19 dataset that has extra CT images with clinical cases that have no COVID-19. For feature selection, the proposed SFS-Guided WOA algorithm is compared in experiments with the original WOA, GWO, GA, PSO, hybrid of PSO and GWO (GWO-PSO), hybrid of GA and GWO (GWO-GA), BA, BBO, MVO, SBO, and FA in terms of average error, average select size, average (mean) fitness, best fitness, worst fitness, and standard deviation fitness. Finally, the proposed voting classifier (PSO-Guided WOA) result is compared with voting WOA, voting GWO, voting GA, and Voting PSO in terms of AUC and MSE. The statistical analysis of Wilcoxon rank-sum, ANOVA, and T-Test shows the superiority of the proposed algorithms. The utilization of each successive phase is aimed to improve the overall accuracy to offer a viable and reliable paradigm in the battle against the spread of COVID-19. A future research direction will be to tune the CNN parameters to increase the overall classification accuracy in case of using other datasets that cannot achieve satisfactory performance. Moreover, the proposed algorithms can be applied to several medical image processing applications that use other imaging modalities.

## References

[1] C.-C. Lai, T.-P. Shih, W.-C. Ko, H.-J. Tang, and P.-R. Hsueh, “Severe acute respiratory syndrome coronavirus 2 (SARS-CoV-2) and coronavirus disease-2019 (COVID-19): The epidemic and the challenges,” Int. J. Antimicrobial Agents, vol. 55, no. 3, Mar. 2020, Art. no. 105924. [Online]. Available: http://www.sciencedirect.com/science/article/pii/S0924857920300674 10.1016/j.ijantimicag.2020.105924 PMC7127800 32081636

[2] C. Wang, P. W. Horby, F. G. Hayden, and G. F. Gao, “A novel coronavirus outbreak of global health concern,” The Lancet, vol. 395, no. 10223, pp. 470–473, 2020, doi: 10.1016/S0140-6736(20)30185-9. PMC7135038 31986257

[3] H. Y. F. Wong, H. Y. S. Lam, A. H.-T. Fong, S. T. Leung, T. W.-Y. Chin, C. S. Y. Lo, M. M.-S. Lui, J. C. Y. Lee, K. W.-H. Chiu, T. Chung, E. Y. P. Lee, E. Y. F. Wan, F. N. I. Hung, T. P. W. Lam, M. Kuo, and M.-Y. Ng, “Frequency and distribution of chest radiographic findings in COVID-19 positive patients,” Radiology, vol. 2019, Mar. 2019, Art. no. 201160, doi: 10.1148/radiol.2020201160. PMC7233401 32216717

[4] S. Cho, S. Lim, C. Kim, S. Wi, T. Kwon, W. S. Youn, S. H. Lee, B. S. Kang, and S. Cho, “Enhancement of soft-tissue contrast in cone-beam CT using an anti-scatter grid with a sparse sampling approach,” Phys. Medica, vol. 70, pp. 1–9, Feb. 2020, doi: 10.1016/j.ejmp.2020.01.004. 31931280

[5] J. Fu, J. Wang, W. Guo, and P. Peng, “Multi-mounted X-Ray cone-beam computed tomography,” Nucl. Instrum. Methods Phys. Res. A, Accel. Spectrom. Detect. Assoc. Equip., vol. 888, pp. 119–125, Oct. 2018. [Online]. Available: http://www.sciencedirect.com/science/article/pii/S0168900218300615

[6] A. Ibrahim, S. Mohammed, and H. A. Ali, “Breast cancer detection and classification using thermography: A review,” in Proc. Int. Conf. Adv. Mach. Learn. Technol. Appl., A. E. Hassanien, M. F. Tolba, M. Elhoseny, and M. Mostafa, Eds. Cham, Switzerland: Springer, 2018, pp. 496–505.

[7] K. Ye, Q. Zhu, M. Li, Y. Lu, and H. Yuan, “A feasibility study of pulmonary nodule detection by ultralow-dose CT with adaptive statistical iterative reconstruction-V technique,” Eur. J. Radiol., vol. 119, Oct. 2019, Art. no. 108652. 10.1016/j.ejrad.2019.108652 31521879

[8] J. Fu, X. Hu, A. Velroyen, M. Bech, M. Jiang, and F. Pfeiffer, “3D algebraic iterative reconstruction for cone-beam X-Ray differential phase-contrast computed tomography,” PLoS ONE, vol. 10, no. 3, Mar. 2015, Art. no. e0117502, doi: 10.1371/journal.pone.0117502. PMC4361763 25775480

[9] B. J. Walker, J. Radtke, G.-H. Chen, K. W. Eliceiri, and T. R. Mackie, “A beam optics study of a modular multi-source X-ray tube for novel computed tomography applications,” Nucl. Instrum. Methods Phys. Res. A, Accel. Spectrom. Detect. Assoc. Equip., vol. 868, pp. 1–9, Oct. 2017. [Online]. Available: http://www.sciencedirect.com/science/article/pii/S0168900217306885

[10] L. T. Campos, F. M. de Jesus, E. A. de Souza Goncalves, and L. A. G. Magalhaes, “Computed tomography X-Ray characterization: A Monte Carlo study,” Radiat. Phys. Chem., vol. 167, Oct. 2020, Art. no. 108359. [Online]. Available: http://www.sciencedirect.com/science/article/pii/S0969806X18313252

[11] M. K. M. Honkanen, H. Matikka, J. T. J. Honkanen, A. Bhattarai, M. W. Grinstaff, A. Joukainen, H. Kröger, J. S. Jurvelin, and J. Töyräs, “Imaging of proteoglycan and water contents in human articular cartilage with full–body CT using dual contrast technique,” J. Orthopaedic Res., vol. 37, no. 5, pp. 1059–1070, 5 2019. [Online]. Available: https://onlinelibrary.wiley.com/doi/abs/10.1002/jor.24256 10.1002/jor.24256 PMC6594070 30816584

[12] E. Montagnon, M. Cerny, A. Cadrin-Chánevert, V. Hamilton, T. Derennes, A. Ilinca, F. Vandenbroucke-Menu, S. Turcotte, S. Kadoury, and A. Tang, “Deep learning workflow in radiology: A primer,” Insights into Imag., vol. 11, no. 1, p. 22, Dec. 2020. 10.1186/s13244-019-0832-5 PMC7010882 32040647

[13] A. Ibrahim, S. Mohammed, H. A. Ali, and S. E. Hussein, “Breast cancer segmentation from thermal images based on chaotic Salp swarm algorithm,” IEEE Access, vol. 8, no. 1, pp. 122121–122134, 2020, doi: 10.1109/access.2020.3007336.

[14] J. Han, D. Zhang, G. Cheng, N. Liu, and D. Xu, “Advanced deep-learning techniques for salient and category-specific object detection: A survey,” IEEE Signal Process. Mag., vol. 35, no. 1, pp. 84–100, Jan. 2018, doi: 10.1109/msp.2017.2749125.

[15] K. Simonyan and A. Zisserman, “Very deep convolutional networks for large-scale image recognition,” 2014, arXiv:1409.1556. [Online]. Available: http://arxiv.org/abs/1409.1556

[16] A. Al-Dhamari, R. Sudirman, and N. H. Mahmood, “Transfer deep learning along with binary support vector machine for abnormal behavior detection,” IEEE Access, vol. 8, pp. 61085–61095, 2020, doi: 10.1109/access.2020.2982906.

[17] S. Yu, L. Xie, L. Liu, and D. Xia, “Learning long-term temporal features with deep neural networks for human action recognition,” IEEE Access, vol. 8, pp. 1840–1850, 2020, doi: 10.1109/access.2019.2962284.

[18] R. Yamashita, M. Nishio, R. K. G. Do, and K. Togashi, “Convolutional neural networks: An overview and application in radiology,” Insights into Imag., vol. 9, no. 4, pp. 611–629, Aug. 2018. 10.1007/s13244-018-0639-9 PMC6108980 29934920

[19] M. M. Fouad, A. I. El-Desouky, R. Al-Hajj, and E.-S. M. El-Kenawy, “Dynamic group-based cooperative optimization algorithm,” IEEE Access, vol. 8, pp. 148378–148403, 2020, doi: 10.1109/access.2020.3015892.

[20] M. A. A. Al-qaness, A. A. Ewees, H. Fan, and M. A. El Aziz, “Optimization method for forecasting confirmed cases of COVID-19 in China,” J. Clin. Med., vol. 9, no. 3, p. 674, Mar. 2020, doi: 10.3390/jcm9030674. PMC7141184 32131537

[21] A. Tharwat, “Parameter investigation of support vector machine classifier with kernel functions,” Knowl. Inf. Syst., vol. 61, no. 3, pp. 1269–1302, Dec. 2019. [Online]. Available: http://www.sciencedirect.com/science/article/pii/S0169260720309664

[22] R. M. Pereira, D. Bertolini, L. O. Teixeira, C. N. Silla, and Y. M. G. Costa, “COVID-19 identification in chest X-ray images on flat and hierarchical classification scenarios,” Comput. Methods Programs Biomed., vol. 194, Oct. 2020, Art. no. 105532. [Online]. Available: http://www.sciencedirect.com/science/article/pii/S0169260720309664 10.1016/j.cmpb.2020.105532 PMC7207172 32446037

[23] S. Jang, Y.-E. Jang, Y.-J. Kim, and H. Yu, “Input initialization for inversion of neural networks using k-nearest neighbor approach,” Inf. Sci., vol. 519, pp. 229–242, 5 2020. [Online]. Available: http://www.sciencedirect.com/science/article/pii/S0020025520300426

[24] L. Breiman, “Random forests,” Mach. Learn., vol. 45, no. 1, pp. 5–32, 2001, doi: 10.1023/a:1010933404324.

[25] S. Mirjalili and A. Lewis, “The whale optimization algorithm,” Adv. Eng. Softw., vol. 95, pp. 51–67, 5 2016.

[26] Q. Al-Tashi, S. J. Abdul Kadir, H. M. Rais, S. Mirjalili, and H. Alhussian, “Binary optimization using hybrid grey wolf optimization for feature selection,” IEEE Access, vol. 7, pp. 39496–39508, 2019.

[27] M. M. Kabir, M. Shahjahan, and K. Murase, “A new local search based hybrid genetic algorithm for feature selection,” Neurocomputing, vol. 74, no. 17, pp. 2914–2928, Oct. 2011. [Online]. Available: http://www.sciencedirect.com/science/article/pii/S0925231211002748

[28] R. Bello, Y. Gomez, A. Nowe, and M. M. Garcia, “Two-step particle swarm optimization to solve the feature selection problem,” in Proc. 7th Int. Conf. Intell. Syst. Design Appl. (ISDA), Oct. 2007, pp. 691–696.

[29] F. A. Şenel, F. Gokçe, A. S. Yuksel, and T. Yigit, “A novel hybrid PSO GWO algorithm for optimization problems,” Eng. Comput., vol. 35, no. 4, pp. 1359–1373, Dec. 2019, doi: 10.1007/s00366-018-0668-5.

[30] I. Karakonstantis and A. Vlachos, “Bat algorithm applied to continuous constrained optimization problems,” J. Inf. Optim. Sci., vol. 5, pp. 1–19, Mar. 2020, doi: 10.1080/02522667.2019.1694740.

[31] D. Simon, “Biogeography-based optimization,” IEEE Trans. Evol. Comput., vol. 12, no. 6, pp. 702–713, Dec. 2008, doi: 10.1109/tevc.2008.919004.

[32] S. Mirjalili, S. M. Mirjalili, and A. Hatamlou, “Multi-verse optimizer: A nature-inspired algorithm for global optimization,” Neural Comput. Appl., vol. 27, no. 2, pp. 495–513, Feb. 2016, doi: 10.1007/s00521-015-1870-7.

[33] S. H. Samareh Moosavi and V. Khatibi Bardsiri, “Satin bowerbird optimizer: A new optimization algorithm to optimize ANFIS for software development effort estimation,” Eng. Appl. Artif. Intell., vol. 60, pp. 1–15, Apr. 2017, doi: 10.1016/j.engappai.2017.01.006.

[34] I. Fister Jr, X.-S. Yang, I. Fister, and J. Brest, “Memetic firefly algorithm for combinatorial optimization,” 2012, arXiv:1204.5165. [Online]. Available: http://arxiv.org/abs/1204.5165

[35] K. Li, Y. Fang, W. Li, C. Pan, P. Qin, Y. Zhong, X. Liu, M. Huang, Y. Liao, and S. Li, “CT image visual quantitative evaluation and clinical classification of coronavirus disease (COVID-19),” Eur. Radiol., vol. 30, no. 8, pp. 4407–4416, Mar. 2020, doi: 10.1007/s00330-020-06817-6. 32215691 PMC7095246

[36] M. Chung, A. Bernheim, X. Mei, N. Zhang, M. Huang, X. Zeng, J. Cui, W. Xu, Y. Yang, Z. A. Fayad, A. Jacobi, K. Li, S. Li, and H. Shan, “CT imaging features of 2019 novel coronavirus (2019-nCoV),” Radiology, vol. 295, no. 1, pp. 202–207, Apr. 2020, doi: 10.1148/radiol.2020200230. 32017661 PMC7194022

[37] R. Yang, X. Li, H. Liu, Y. Zhen, X. Zhang, Q. Xiong, Y. Luo, C. Gao, and W. Zeng, “Chest CT severity score: An imaging tool for assessing severe COVID-19,” Radiol., Cardiothoracic Imag., vol. 2, no. 2, Apr. 2020, Art. no. e200047, doi: 10.1148/ryct.2020200047. PMC7233443 33778560

[38] H. Shi, X. Han, N. Jiang, Y. Cao, O. Alwalid, J. Gu, Y. Fan, and C. Zheng, “Radiological findings from 81 patients with COVID-19 pneumonia in wuhan, China: A descriptive study,” Lancet Infectious Diseases, vol. 20, no. 4, pp. 425–434, Apr. 2020. 32105637 10.1016/S1473-3099(20)30086-4 PMC7159053

[39] H. X. Bai, B. Hsieh, Z. Xiong, K. Halsey, J. W. Choi, T. M. L. Tran, I. Pan, L.-B. Shi, D.-C. Wang, J. Mei, X.-L. Jiang, Q.-H. Zeng, T. K. Egglin, P.-F. Hu, S. Agarwal, F. Xie, S. Li, T. Healey, M. K. Atalay, and W.-H. Liao, “Performance of radiologists in differentiating COVID-19 from viral pneumonia on chest CT,” Radiology, vol. 12, Mar. 2020, Art. no. 200823, doi: 10.1148/radiol.2020200823. PMC7233414 32155105

[40] F. Chua, “The role of CT in case ascertainment and management of COVID-19 pneumonia in the UK: Insights from high-incidence regions,” Lancet Respiratory Med., vol. 8, no. 5, pp. 438–440, 5 2020, doi: 10.1016/s2213-2600(20)30132-6. PMC7104153 32220663

[41] X. Wu, H. Hui, M. Niu, L. Li, L. Wang, B. He, X. Yang, L. Li, H. Li, J. Tian, and Y. Zha, “Deep learning-based multi-view fusion model for screening 2019 novel coronavirus pneumonia: A multicentre study,” Eur. J. Radiol., vol. 128, Jul. 2020, Art. no. 109041. [Online]. Available: http://www.sciencedirect.com/science/article/pii/S0720048X20302308 10.1016/j.ejrad.2020.109041 PMC7198437 32408222

[42] A. A. Ardakani, A. R. Kanafi, U. R. Acharya, N. Khadem, and A. Mohammadi, “Application of deep learning technique to manage COVID-19 in routine clinical practice using CT images: Results of 10 convolutional neural networks,” Comput. Biol. Med., vol. 121, Jun. 2020, Art. no. 103795. [Online]. Available: http://www.sciencedirect.com/science/article/pii/S0010482520301645 10.1016/j.compbiomed.2020.103795 PMC7190523 32568676

[43] , “Clinically applicable AI system for accurate diagnosis, quantitative measurements, and prognosis of COVID-19 pneumonia using computed tomography,” Cell, vol. 181, no. 6, pp. 1423–1433, Jun. 2020, doi: 10.1016/j.cell.2020.04.045. 32416069 PMC7196900

[44] H. Panwar, P. K. Gupta, M. K. Siddiqui, R. Morales-Menendez, and V. Singh, “Application of deep learning for fast detection of COVID-19 in X-rays using nCOVnet,” Chaos, Solitons Fractals, vol. 138, Sep. 2020, Art. no. 109944. [Online]. Available: http://www.sciencedirect.com/science/article/pii/S096007792030343X 10.1016/j.chaos.2020.109944 PMC7254021 32536759

[45] C. Butt, J. Gill, D. Chun, and B. A. Babu, “Deep learning system to screen coronavirus disease 2019 pneumonia,” Int. J. Speech Technol., vol. 1, p. 22, Apr. 2020, doi: 10.1007/s10489-020-01714-3. PMC7175452 38624372

[46] M. Nour, Z. Cömert, and K. Polat, “A novel medical diagnosis model for COVID-19 infection detection based on deep features and Bayesian optimization,” Appl. Soft Comput., vol. 46, Jul. 2020, Art. no. 106580, doi: 10.1016/j.asoc.2020.106580. PMC7385069 32837453

[47] S. Hu, Y. Gao, Z. Niu, Y. Jiang, L. Li, X. Xiao, M. Wang, E. F. Fang, W. Menpes-Smith, J. Xia, H. Ye, and G. Yang, “Weakly supervised deep learning for COVID-19 infection detection and classification from CT images,” IEEE Access, vol. 8, pp. 118869–118883, 2020, doi: 10.1109/access.2020.3005510.

[48] M. A. Elaziz, K. M. Hosny, A. Salah, M. M. Darwish, S. Lu, and A. T. Sahlol, “New machine learning method for image-based diagnosis of COVID-19,” PLoS ONE, vol. 15, no. 6, Jun. 2020, Art. no. e0235187, doi: 10.1371/journal.pone.0235187. PMC7319603 32589673

[49] J. Zhao, Y. Zhang, X. He, and P. Xie, “COVID-CT-Dataset: A CT Scan Dataset about COVID-19,” 2020, arXiv:2003.13865. [Online]. Available: https://arxiv.org/abs/2003.13865

[50] W. Naudé, “Artificial intelligence vs COVID-19: Limitations, constraints and pitfalls,” AI Soc., vol. 35, no. 3, pp. 761–765, Sep. 2020, doi: 10.1109/access.2019.2955983. PMC7186767 32346223

[51] E. M. Hassib, A. I. El-Desouky, E.-S. M. El-Kenawy, and S. M. El-Ghamrawy, “An imbalanced big data mining framework for improving optimization algorithms performance,” IEEE Access, vol. 7, pp. 170774–170795, 2019, doi: 10.1109/access.2019.2955983.

[52] E. M. Hassib, A. I. El-Desouky, L. M. Labib, and E.-S.-M. El-kenawy, “WOA + BRNN: An imbalanced big data classification framework using whale optimization and deep neural network,” Soft Comput., vol. 24, no. 8, pp. 5573–5592, Mar. 2019, doi: 10.1007/s00500-019-03901-y.

[53] S. Mirjalili, S. M. Mirjalili, S. Saremi, and S. Mirjalili, Whale Optim. Algorithm: Theory, Literature Rev., Appl. Designing Photonic Crystal Filters. Cham: Springer International Publishing, 2020, pp. 219–238, doi: 10.1007/978-3-030-12127-3_13.

[54] E. Cuevas, F. Fausto, and A. González, Metaheuristics and Swarm Methods: A Discussion on Their Performance and Applications. Cham, Switzerland: Springer, 2020, pp. 43–67, doi: 10.1007/978-3-030-16339-6_2.

[55] F. Fausto, A. Reyna-Orta, E. Cuevas, Á.G. Andrade, and M. Perez-Cisneros, “From ants to whales: Metaheuristics for all tastes,” Artif. Intell. Rev., vol. 53, no. 1, pp. 753–810, Jan. 2020.

[56] H. Salimi, “Stochastic fractal search: A powerful metaheuristic algorithm,” Knowl.-Based Syst., vol. 75, pp. 1–18, Oct. 2015. [Online]. Available: http://www.sciencedirect.com/science/article/pii/S0950705114002822

[57] E.-S. M. El-Kenawy, M. M. Eid, M. Saber, and A. Ibrahim, “MbGWO-SFS: Modified binary grey wolf optimizer based on stochastic fractal search for feature selection,” IEEE Access, vol. 8, no. 1, pp. 107635–107649, 2020, doi: 10.1109/access.2020.3001151.

[58] E.-S. El-Kenawy and M. Eid, “Hybrid gray wolf and particle swarm optimization for feature selection,” Int. J. Innov. Comput., Inf. Control, vol. 16, no. 3, pp. 831–844, 2020.

[59] A. Ibrahim, M. Noshy, H. A. Ali, and M. Badawy, “PAPSO: A power-aware VM placement technique based on particle swarm optimization,” IEEE Access, vol. 8, pp. 81747–81764, 2020, doi: 10.1109/access.2020.2990828.

[60] T. Gaber, A. Tharwat, A. Ibrahim, V. Snael, and A. E. Hassanien, “Human thermal face recognition based on random linear oracle (RLO) ensembles,” in Proc. Int. Conf. Intell. Netw. Collaborative Syst., Sep. 2015, pp. 1–5, doi: 10.1109/incos.2015.67.

[61] A. Ibrahim, A. Tharwat, T. Gaber, and A. E. Hassanien, “Optimized superpixel and AdaBoost classifier for human thermal face recognition,” Signal, Image Video Process., vol. 12, no. 4, pp. 711–719, Nov. 2017, doi: 10.1007/s11760-017-1212-6.

